# Biomarkers of Neurobiologic Recovery in Adults With Sport-Related Concussion

**DOI:** 10.1001/jamanetworkopen.2024.15983

**Published:** 2024-06-07

**Authors:** William T. O’Brien, Gershon Spitz, Becca Xie, Brendan P. Major, Steven Mutimer, Lauren P. Giesler, Jesse Bain, Lauren J. Evans, Beatriz Duarte Martins, Stefan Piantella, Afizu Alhassan, Shelby Brady, David Cappellari, Vincenzo Somma, Thomas McColl, Georgia F. Symons, Tenae Gore, Matthew Sun, Timothy Kuek, Seamus Horan, Michael Bei, Jennie L. Ponsford, Catherine Willmott, Jonathan Reyes, Nicholas J. Ashton, Henrik Zetterberg, Biswadev Mitra, Terence J. O’Brien, Sandy R. Shultz, Stuart J. McDonald

**Affiliations:** 1 Department of Neuroscience, Monash University, Melbourne, Victoria, Australia; 2 Monash-Epworth Rehabilitation Research Centre, School of Psychological Sciences, Monash University, Melbourne, Victoria, Australia; 3 Australian Football League, Melbourne, Victoria, Australia; 4 Department of Psychiatry and Neurochemistry, Institute of Neuroscience and Physiology, Sahlgrenska Academy, University of Gothenburg, Mölndal, Sweden; 5 King’s College London, Institute of Psychiatry, Psychology and Neuroscience, Maurice Wohl Institute Clinical Neuroscience Institute, London, United Kingdom; 6 NIHR Biomedical Research Centre for Mental Health and Biomedical Research Unit for Dementia at South London and Maudsley NHS Foundation, London, United Kingdom; 7 Centre for Age-Related Medicine, Stavanger University Hospital, Stavanger, Norway; 8 Clinical Neurochemistry Laboratory, Sahlgrenska University Hospital, Mölndal, Sweden; 9 Department of Neurodegenerative Disease, University College London Institute of Neurology, Queen Square, London, United Kingdom; 10 UK Dementia Research Institute at University College London, London, United Kingdom; 11 Hong Kong Center for Neurodegenerative Diseases, Hong Kong, Hong Kong SAR, China; 12 Wisconsin Alzheimer’s Disease Research Center, University of Wisconsin School of Medicine and Public Health, University of Wisconsin-Madison; 13 Emergency & Trauma Centre, The Alfred Hospital, Australia; 14 School of Public Health & Preventive Medicine, Monash University, Melbourne, Victoria, Australia; 15 Department of Neurology, The Alfred Hospital, Melbourne, Victoria, Australia; 16 Department of Medicine, Royal Melbourne Hospital, The University of Melbourne, Parkville, Victoria, Australia; 17 Health Sciences, Vancouver Island University, Nanaimo, British Columbia, Canada

## Abstract

**Importance:**

Sport-related concussion (SRC), a form of mild traumatic brain injury, is a prevalent occurrence in collision sports. There are no well-established approaches for tracking neurobiologic recovery after SRC.

**Objective:**

To examine the levels of serum glial fibrillary acidic protein (GFAP) and neurofilament light (NfL) in Australian football athletes who experience SRC.

**Design, Setting, and Participants:**

A cohort study recruiting from April 10, 2021, to September 17, 2022, was conducted through the Victorian Amateur Football Association, Melbourne, Australia. Participants included adult Australian football players with or without SRC. Data analysis was performed from May 26, 2023, to March 27, 2024.

**Exposure:**

Sport-related concussion, defined as at least 1 observable sign and/or 2 or more symptoms.

**Main Outcomes and Measures:**

Primary outcomes were serum GFAP and NfL levels at 24 hours, and 1, 2, 4, 6, 8, 12, and 26 weeks. Secondary outcomes were symptoms, cognitive performance, and return to training times.

**Results:**

Eighty-one individuals with SRC (median age, 22.8 [IQR, 21.3-26.0] years; 89% male) and 56 control individuals (median age, 24.6 [IQR, 22.4-27.3] years; 96% male) completed a total of 945 of 1057 eligible testing sessions. Compared with control participants, those with SRC exhibited higher GFAP levels at 24 hours (mean difference [MD] in natural log, pg/mL, 0.66 [95% CI, 0.50-0.82]) and 4 weeks (MD, 0.17 [95% CI, 0.02-0.32]), and NfL from 1 to 12 weeks (1-week MD, 0.31 [95% CI, 0.12-0.51]; 2-week MD, 0.38 [95% CI, 0.19-0.58]; 4-week MD, 0.31 [95% CI, 0.12-0.51]; 6-week MD, 0.27 [95% CI, 0.07-0.47]; 8-week MD, 0.36 [95% CI, 0.15-0.56]; and 12-week MD, 0.25 [95% CI, 0.04-0.46]). Growth mixture modeling identified 2 GFAP subgroups: extreme prolonged (16%) and moderate transient (84%). For NfL, 3 subgroups were identified: extreme prolonged (7%), moderate prolonged (15%), and minimal or no change (78%). Individuals with SRC who reported loss of consciousness (LOC) (33% of SRC cases) had higher GFAP at 24 hours (MD, 1.01 [95% CI, 0.77-1.24]), 1 week (MD, 0.27 [95% CI, 0.06-0.49]), 2 weeks (MD, 0.21 [95% CI, 0.004-0.42]) and 4 weeks (MD, 0.34 [95% CI, 0.13-0.55]), and higher NfL from 1 week to 12 weeks (1-week MD, 0.73 [95% CI, 0.42-1.03]; 2-week MD, 0.91 [95% CI, 0.61-1.21]; 4-week MD, 0.90 [95% CI, 0.59-1.20]; 6-week MD, 0.81 [95% CI, 0.50-1.13]; 8-week MD, 0.73 [95% CI, 0.42-1.04]; and 12-week MD, 0.54 [95% CI, 0.22-0.85]) compared with SRC participants without LOC. Return to training times were longer in the GFAP extreme compared with moderate subgroup (incident rate ratio [IRR], 1.99 [95% CI, 1.69-2.34]; NfL extreme (IRR, 3.24 [95% CI, 2.63-3.97]) and moderate (IRR, 1.43 [95% CI, 1.18-1.72]) subgroups compared with the minimal subgroup, and for individuals with LOC compared with those without LOC (IRR, 1.65 [95% CI, 1.41-1.93]).

**Conclusions and Relevance:**

In this cohort study, a subset of SRC cases, particularly those with LOC, showed heightened and prolonged increases in GFAP and NfL levels, that persisted for at least 4 weeks. These findings suggest that serial biomarker measurement could identify such cases, guiding return to play decisions based on neurobiologic recovery. While further investigation is warranted, the association between prolonged biomarker elevations and LOC may support the use of more conservative return to play timelines for athletes with this clinical feature.

## Key Points

**Question:**

Do distinct trajectory subgroups exist in the serum levels of glial fibrillary acidic protein (GFAP) and neurofilament light (NfL) following sport-related concussion?

**Findings:**

In this cohort study of 81 individuals with sport-related concussion, in a subset of cases, increases in GFAP and NfL levels were substantial and persisted for at least 4 weeks. Individuals in these extreme biomarker subgroups were more likely to have experienced loss of consciousness (LOC) and longer to return to training times.

**Meaning:**

The findings of this study suggest the utility of serial measurements of GFAP and NfL to track neurobiologic recovery, with the association between LOC and extended biomarker elevations supporting the use of LOC for informing more conservative return-to-play timelines.

## Teaser

This cohort study examines the levels of serum glial fibrillary acidic protein and neurofilament light in athletes in Australia who experience sport-related concussion.

## Introduction

Sport-related concussion (SRC), a form of mild traumatic brain injury (mTBI), is a prevalent occurrence in collision sports. This is exemplified by Australian football, with an annual participation exceeding 500 000 individuals and an incidence of 6 to 10 SRCs per 1000 player match-hours.^1,2,3^ The ramifications of SRC may transcend short-term symptoms, with evidence linking repeated SRCs to chronic neurologic consequences.^4,5,6,7^

A factor likely increasing the risk of cumulative and chronic consequences is repeated SRCs in short succession.^8,9^ Multiple sporting associations have recently extended mandatory minimum return to play times after SRC, generally ranging from 11 to 21 days depending on the sport and level of competition.^10,11^ While return to play decisions after this period should be contingent on symptom resolution, progression through a graded loading program, and medical clearance,^12,13^ there is a common misconception that this timeframe universally defines recovery. This concern is pronounced in community sport, where medical guidance may be limited. Adding to the complexity, symptoms are subjective and athletes may not disclose them.^14,15^ Neurobiologic disruptions can persist beyond symptoms,^16,17,18^ and such changes may contribute to vulnerability to repeated SRC.^19,20,21^ Recognizing these challenges supports individualized return to play approaches, necessitating the development of objective tools for personalized assessment of both clinical and neurobiologic recovery.^12,22^

Venous blood levels of glial fibrillary acidic protein (GFAP) and neurofilament light (NfL) have emerged as candidate biomarkers of mTBI and SRC.^16,17,23,24^ These markers exhibit high specificity to astroglial injury for GFAP and axonal injury with NfL, with both forms of injury potentially progressing post impact.^25,26^ The magnitude and timing of GFAP and NfL peak, decrease, and resolution in blood are likely critical aspects that could inform understanding of brain injury progression and recovery. For example, considering that the circulating half-life of GFAP is relatively short (approximately 24-48 hours^27^) compared with NfL (approximately 3 weeks^28,29^), resolution of GFAP and a decrease in NfL could signify the cessation of brain release of these damage-associated proteins during SRC recovery.

To date, studies have found heterogeneity in GFAP and NfL profiles after SRC^30,31^ and have primarily focused on the first 2 weeks or featured large time-bins and attrition. A deeper understanding of biomarker trajectories and heterogeneity is required to improve comprehension of typical and atypical timeframes for neurobiologic recovery. Moreover, although increased NfL levels have been associated with longer return to play times,^32^ how GFAP and NfL trajectories relate to symptoms, cognitive performance and return to training (RTT) remains unknown. In addition, while there is preliminary evidence that GFAP and NfL levels may be higher in SRC cases involving loss of consciousness (LOC),^24^ it is unknown whether LOC alone can predict the extent and duration of neurobiologic changes.

In this study of Australian football players, we aimed to construct a comprehensive temporal profile for serum GFAP and NfL after SRC and identify subgroups with distinct trajectories. In addition, we investigated GFAP and NfL trajectories and their association with the presence of LOC, extent and duration of symptoms and cognitive disturbances, and RTT timeframes.

## Methods

### Recruitment

This cohort study followed the Strengthening the Reporting of Observational Studies in Epidemiology (STROBE) reporting guideline. Procedures were approved by the Monash University Human Research Ethics Committee. Written informed consent to participate was provided by all participants at the first testing session.

Between April 10, 2021, and September 17, 2022, players with suspected SRC (n = 146) were identified during Australian football matches and training by Victorian Amateur Football Association club medical staff (physicians, physiotherapists, or sport trainers) from 71 teams (55 male, 16 female). Players without any injuries (ie, healthy controls) or a musculoskeletal injury were sought from a selection of teams across each level. Inclusion criteria for SRC closely aligned with the 2017 Concussion in Sport Group definition. Specific criteria were a biomechanically plausible mechanism of injury and at least 1 observable sign and/or 2 or more symptoms using the Sports Concussion Assessment Tool 5 or Concussion Recognition Tool 5. Observable signs were (1) lying motionless on the playing surface; (2) balance, gait difficulties, motor incoordination, stumbling, or slow labored movements; (3) disorientation/confusion or inability to respond to questions; and (4) blank/vacant look. Symptoms were reported as headache, pressure in head, neck pain, nausea and vomiting, dizziness, blurred vision, balance problems, sensitivity to light, sensitivity to noise, feeling slowed down, feeling like in a fog, do not feel right, difficulty concentrating, difficulty remembering, fatigue/low energy, confusion, drowsiness, more emotional, irritability, sadness, nervous/anxious, and trouble falling asleep. Inclusion criteria for control subgroups (collapsed for primary analysis) were musculoskeletal injury predicted to require 1 to 3 missed matches, and for healthy controls, match completion without injury. To minimize the potential confounding factor of prior concussions associated with biomarker profiles, participants were excluded if they reported a concussion within 6 months, except where SRC participants sustained a second SRC within the study period (n = 3, each invited to restart study; 1 of these restarted) and during analysis had strong evidence of nonelevated and stable GFAP and NfL levels prior to the second SRC (n = 1). Additional exclusion criteria were a history of moderate to severe TBI, a medical history that may contribute to neurologic impairment, or current major musculoskeletal injury. A recruitment flowchart is shown in eFigure 1 in Supplement 1.

### Data Collection

Sessions were conducted at 24 hours, and 1, 2, 4, 6, 8, 12, and 26 weeks post injury or match. Of the possible 1096 sessions, 39 were cancelled due to ineligibility (ie, concussion or major musculoskeletal injury). Of the 1057 eligible data collections, 945 were completed. Collections occurred within narrow time-bins post injury or match (eg, 24-hour median, 25.0 [IQR, 22.3-28.8] hours) (eTable 1 in Supplement 1). Demographic characteristics (sex, race, age, years of education, height, weight, and concussion history) were self-reported via written survey at the first visit. Race was collected to characterize the cohort for comparison with previous and future research. White and Caucasian were collapsed into a single category. All other responses were reported verbatim. The Rivermead Post Concussion Questionnaire was used to evaluate 16 symptoms on a scale from 0 (not a problem at all) to 4 (a severe problem).^33^

Cognition was assessed with Cogstate,^34^ featuring 4 tasks: Detection (psychomotor speed), Identification (attention), One Card Learning (visual learning), and One Back (working memory). Detection, Identification, and One Back tasks measured reaction latency, while One Card Learning assessed accuracy. Questionnaires collected information on demographics, medical, and sporting history, SRC and musculoskeletal injury details (eg, self-reported LOC), RTT (ie, self-reported noncontact or contact), and return to matches.

### Biomarker Quantification

Venous blood was collected into serum separator tubes and centrifuged at 1500 g at 0.5 to 2 hours post collection. Serum was stored at −80 °C. Serum GFAP and NfL levels were quantified (Simoa HD-X Analyzer, using Neurology 2-Plex B assays; Quanterix). The mean duplicate coefficient of variation was 6.86% for GFAP and 6.42% for NfL. Samples with a coefficient of variation greater than 30% were retested, alongside all samples from the individual. Control sample coefficient of variation was 5.25% for GFAP and 8.71% for NfL.

### Statistical Analysis

Data analysis was conducted from May 26, 2023, to March 27, 2024. Demographic, medical, and sporting history measures were compared using Fisher exact and Wilcoxon rank sum tests. Biomarker subgroups were identified using growth mixture modeling (lcmm package) with fixed effects of age and body mass index. Fixed and random slopes, and nonlinearity of trajectories were tested using exponents 1 to 4. Model fit statistics are provided in eTable 2 and eTable 3 in Supplement 1. Longitudinal data were analyzed with linear mixed models (lme4 package) with age and body mass index as covariates. Fixed and random slopes, and nonlinearity of trajectories were tested using exponents 1 to 7. Akaike information criteria, bayesian information criteria, and simplest model criteria were used to determine appropriate models. Six participants without a 24-hour sample were excluded from the GFAP class analysis (due to the hypothesized peak level of GFAP at 24 hours). All participants were included in all other analyses, regardless of missing time points, as growth mixture modeling and linear mixed models are compatible with missing data. Post hoc analyses were performed with false discovery rate corrections where appropriate (emmeans package). Return to training time was analyzed with a generalized linear model (glm package). Analyses were performed with an adjusted significance threshold of *P* < .05 (2-sided). Power calculations were based on a study comparing acute GFAP in athletes with and without SRC.^24^ With an α level of .05 and power of 0.80, we determined that a sample size of 32 was required. Statistical analysis was performed with R Studio, version 4.2.2 (R Foundation for Statistical Computing).

## Results

### Participant and Injury Characteristics

A total of 130 individual athletes participated, with a median age of 23.2 (IQR, 21.4-26.5) years; 92.3% were male, 1 (0.8%) African, 1 (0.8%) Asian, 1 (0.8%) Indigenous Australian, 121 (98%) White, and 3 Not Answered. With 9 individuals participating twice (eTable 4 in Supplement 1), group sizes were 81 for SRC (median age, 22.8 [IQR, 21.3-26.0] years; 72 [89%] male, 9 [11%] female) and 56 for controls (median age, 24.6 [IQR, 22.4-27.3] years; 54 [96%] male, 2 [4%] female). The control group included 15 individuals with musculoskeletal injury (15 male, 0 female) and 41 healthy individuals (39 male, 2 female). Analyses between the healthy controls and musculoskeletal injury group revealed no substantial biomarker differences (eFigure 2 and eTable 5 in Supplement 1). As such, these were condensed into 1 control group. The SRC and control groups were comparable for all variables except history of concussion (Table 1).

**Table 1.** Participant Demographic and SRC Injury Characteristics^a^

Variable	Participants, No. (%)		*P* value
	SRC (n = 81)	Control (n = 56)	
Sex			
Male	72 (89)	54 (96)	.20^b^
Female	9 (11)	2 (4)	
Race and ethnicity			
African	0	1 (1)	
Asian	1 (1)	0	
Indigenous Australian	1 (1)	0	
White	74 (97)	47 (98)	
No response	5	8	
Age, median (IQR), y	22.8 (21.3-26.0)	24.6 (22.4-27.3)	.10^c^
BMI, median (IQR)	23.72 (22.5 24.9)	24.65 (23.2-25.6)	.08^c^
Years of collision sport, median (IQR)	13.0 (10.5-17.0)	14.0 (10.8-17.0)	.50^c^
History of concussion	56 (69)	29 (52)	.04^b^
Matches played after recruitment, median (IQR)	4.0 (1.0-7.0)	4.0 (2.0-8.0)	.40^c^
CNS-affecting medications	4 (4.9)	2 (3.6)	NA
Antidepressant	2 (2.5)	1(1.8)	NA
CNS stimulant	2 (2.5)	0	NA
Antipsychotic	0	1 (1.8)	NA
Non–CNS-affecting medications	15 (18.5)	6 (10.7)	NA
Anti-inflammatories	4 (4.9)	2 (3.6)	NA
Loss of consciousness			
Yes	27 (33)	NA	NA
No	48 (59)	NA	NA
Unsure	6 (7.4)	NA	NA
Memory impairment			
Yes	36 (44.4)	NA	NA
No	45 (55.6)	NA	NA
Return to training, median (IQR), d	9 (5-12)	NA	NA
Return to match, median (IQR), d	14 (14-21)	NA	NA

Abbreviations: BMI, body mass index (calculated as weight in kilograms squared); CNS, central nervous system; NA, not applicable; SRC, sport-related concussion.

^a^ One control participant had missing data for matches played after recruitment, 15 SRC participants had missing data for return to training, and 29 SRC participants had missing data for return to match (primarily due to end of season, COVID-19 lockdowns, or failure to return to matches).

^b^ Fisher exact test.

^c^ Wilcoxon rank sum test.

### Symptoms and Cognitive Performance

Players with SRC had higher symptom scores at 24 hours, 1 week, and 2 weeks compared with control players (eFigure 3 in Supplement 1). For Cogstate, Detection latency was decreased in the SRC group compared with the control group at 26 weeks, whereas One Back latency was increased in the SRC group at 24 hours and 1 week. Results are detailed in eFigure 4 and eTable 6 in Supplement 1.

### Biomarker Levels

**Figure 1.**
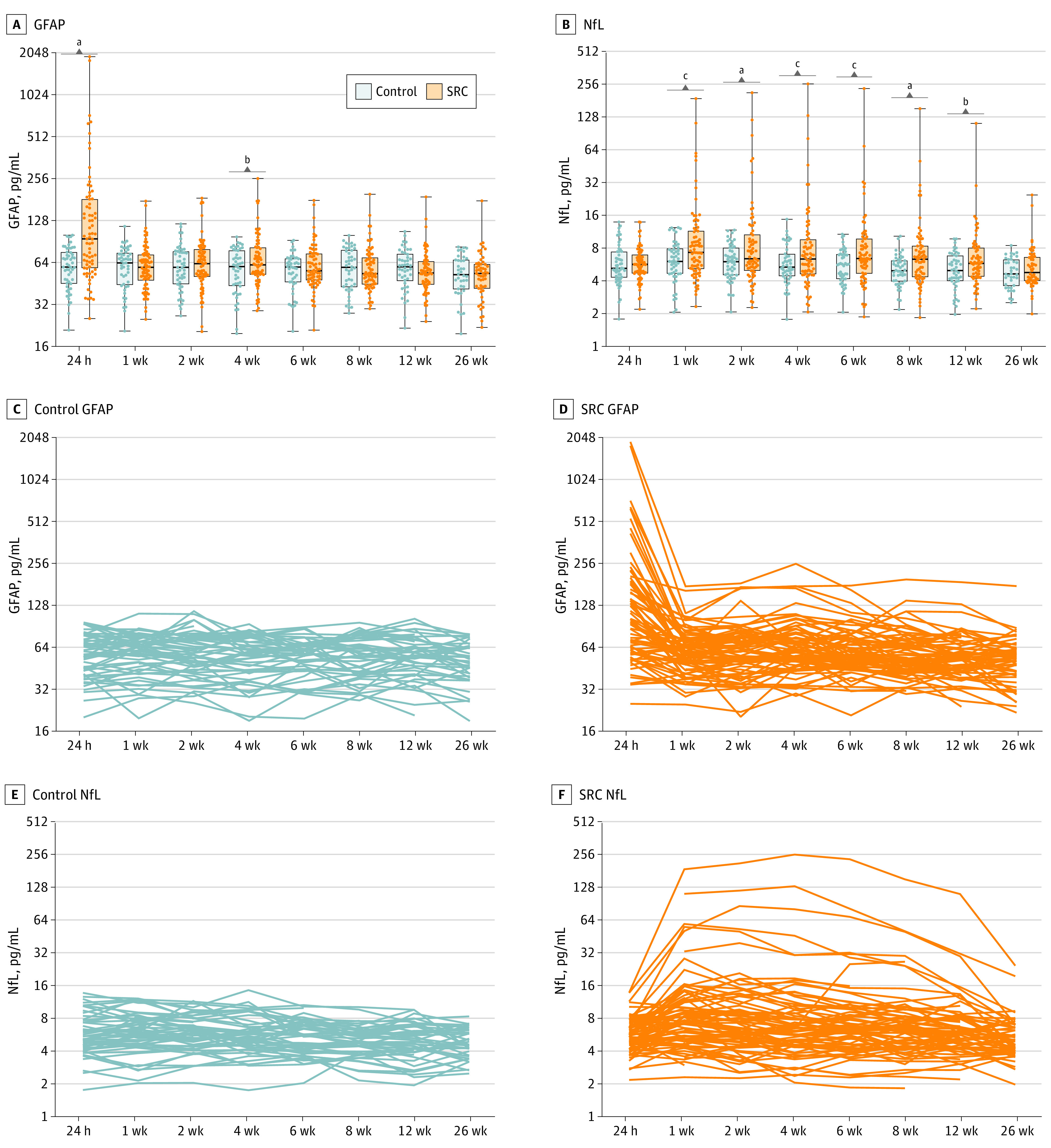
The Profiles of Serum Glial Fibrillary Acidic Protein (GFAP) and Neurofilament Light (NfL) Levels After Sport-Related Concussion At a group level, serum GFAP levels were significantly increased in the sport-related concussion (SRC) participants vs control participants at 24 hours and 4 weeks (A). Serum NfL levels were increased at 1 week, 2 weeks, 4 weeks, 6 weeks, 8 weeks, and 12 weeks (B). While the control group had a relatively consistent profile of GFAP over time (C), the SRC group showed heterogeneity in magnitude of these changes, with some participants appearing to have a secondary increase at approximately 4 weeks (D). Similarly for serum NfL, a comparatively consistent profile was observed in control participants (E), whereas in the SRC participants, there was clear heterogeneity in the extent and timing of peak NfL levels (F). Box plots show the minimum, lower quartile, median, upper quartile, and maximum value. ^a^*P* < .001. ^b^*P* < .05. ^c^*P* < .01.

Serum GFAP levels were higher in SRC participants at 24 hours (mean difference [MD], pg/mL, 0.66; 95% CI, 0.50-0.82) and 4 weeks (MD, 0.17; 95% CI, 0.02-0.32) compared with control participants (Figure 1A). Furthermore, higher NfL levels were found in the SRC group compared with the control group from 1 week to 12 weeks (1-week MD, 0.31; 95% CI, 0.12-0.51; 2-week MD, 0.38; 95% CI, 0.19-0.58; 4-week MD, 0.31; 95% CI, 0.12-0.51; 6-week MD, 0.27; 95% CI, 0.07-0.47; 8-week MD, 0.36; 95% CI, 0.15-0.56; 12-week MD, 0.25; 95% CI, 0.04-0.46]) (Figure 1B). Spaghetti plots (Figure 1, C-F) revealed heterogeneity in SRC profiles. See eTable 6 in Supplement 1 for full results.

### SRC Biomarker Trajectories

**Figure 2.**
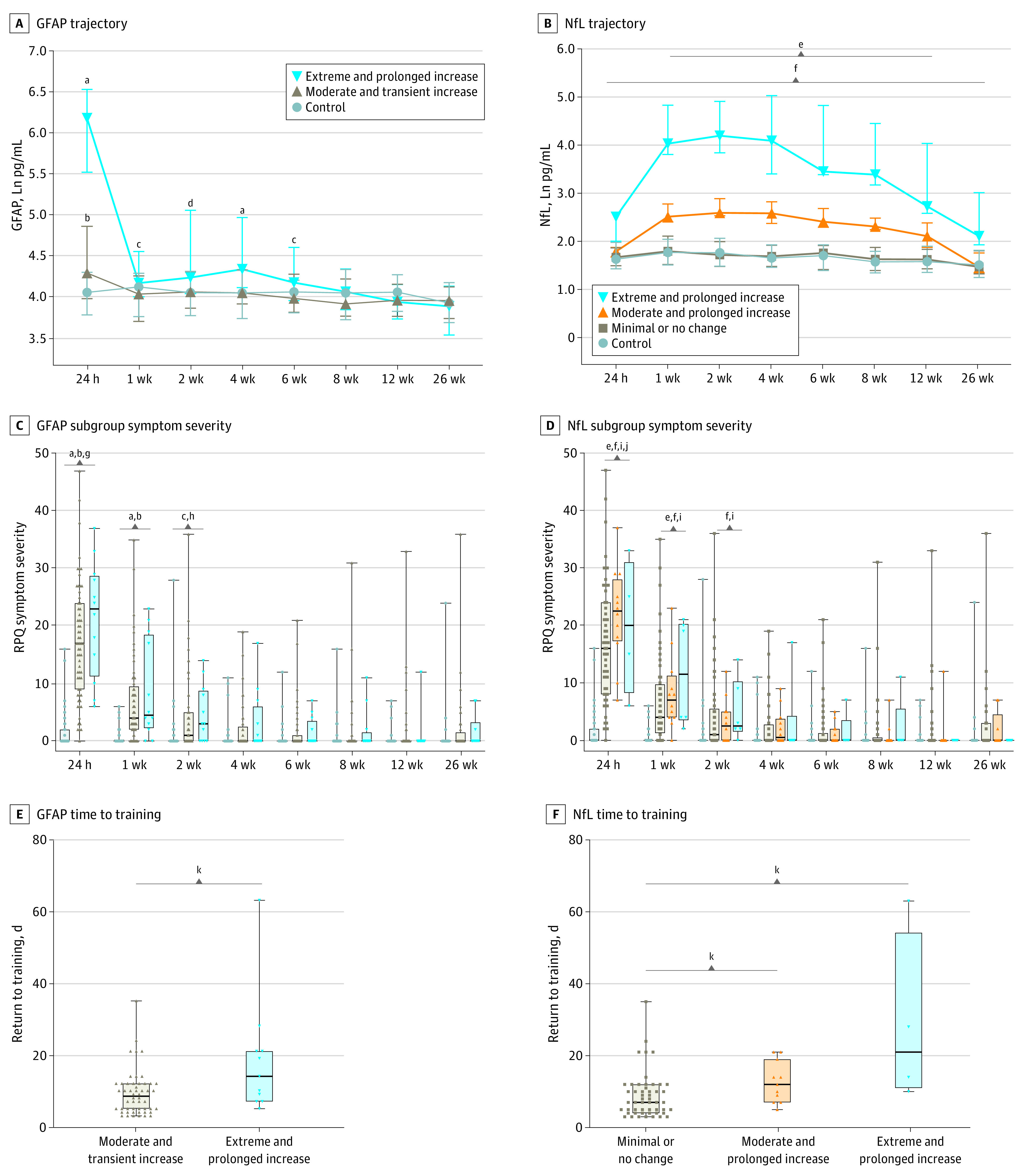
Glial Fibrillary Acidic Protein (GFAP) and Neurofilament Light (NfL) Trajectory Subgroups: Biomarker Profiles, Symptoms and Return to Training Time After Sport-Related Concussion (SRC) Symbols and error bars in A and B represent the median and IQR. Data for C, D, E, and F are presented using box plots showing the minimum, lower quartile, median, upper quartile, and maximum value. ^a^*P* < .001 for the extreme GFAP subgroup vs control participants. ^b^*P* < .001 for the moderate GFAP subgroup vs control participants. ^c^*P* < .05 for the extreme GFAP subgroup vs control participants. ^d^*P* < .01 for the extreme GFAP subgroup vs control participants. ^e^*P* < .05 for the moderate NfL subgroup vs control participants. ^f^*P* < .05 for the extreme NfL subgroup vs control participants. ^g^*P* < .05 for the extreme GFAP subgroup vs moderate GFAP subgroup. ^h^*P* < .05 for the moderate GFAP subgroup vs control participants. ^i^*P* < .05 for the minimal or no change in NfL subgroup vs control participants. ^j^*P* < .05 for the moderate NfL subgroup vs the minimal or no change in NfL subgroup. ^k^*P* < .001.

For GFAP (Figure 2A), 2 distinct trajectory subgroups were identified: one with an extreme and prolonged increase (12 of 75 cases [16.0%]) and another with a moderate and transient increase (63 of 75 cases [84.0%]). Comparison of the 2 subgroups with controls revealed that the extreme subgroup had higher GFAP levels from 24 hours to 6 weeks, whereas the moderate subgroup showed higher levels compared with control participants at 24 hours only. For NfL (Figure 2B), 3 subgroups were found: one with an extreme and prolonged increase (6 of 81 [7.4%]), another with a moderate and prolonged increase (12 of 81 [14.8%]), and a third with minimal no increase (63 of 81 [77.8%]). The extreme and prolonged NfL subgroups had higher levels at all time points compared with control participants. The moderate NfL subgroup had elevated levels from 1 week to 12 weeks. The minimal NfL subgroup had levels no different from control participants. eTable 7 and eTable 8 in Supplement 1 provide full results. A secondary analysis on the timing of peak NfL levels in athletes in the moderate or extreme NfL subgroups who completed all time points out to 12 weeks (n = 14 of 18) showed that 5 participants had peak NfL levels at 1 week, 5 at 2 weeks, and 4 at 4 weeks.

### Biomarker Trajectory Associations With LOC, Symptoms, Cognition, and RTT

Compared with control participants, moderate and extreme GFAP and NfL subgroups had greater symptom severity at 24 hours, 1 week, and 2 weeks (moderate NfL not significant at 2 weeks). Within biomarker comparisons revealed higher 24-hour symptom severity for the GFAP extreme compared with moderate subgroup, and for the moderate NfL compared with the minimal or no change subgroup (Figure 2C and Figure 2D). Cognitive assessment (Cogstate) results for biomarker subgroups are provided in eTable 7, eTable 8, and eFigure 5 in Supplement 1.

We next investigated the association between RTT times with both biomarker subgroup and presence/absence of LOC. The GFAP extreme subgroup had higher RTT times compared with the moderate subgroup (incident rate ratio [IRR], 1.99; 95% CI, 1.69-2.34) (Figure 2E). For NfL, the moderate (IRR, 1.43; 95% CI, 1.18-1.72) and extreme (IRR, 3.24; 95% CI, 2.63-3.97) subgroups reported a greater RTT time than the minimal or no change subgroup (Figure 2F). Full results are available in eTable 9 in Supplement 1. The proportion of individuals who experienced LOC in the extreme GFAP (11 of 12 [91.7%]), and extreme (6 of 6 [100%]) and moderate (8 of 12 ]66.7%]) NfL subgroups was substantially higher than the moderate GFAP (14 of 63 [22.2%]) and minimal or no change NfL subgroups (13 of 63 [20.6%]) (24-hour MD, 1.01; 95% CI, 0.77-1.24; 1-week MD, 0.27; 95% CI, 0.06-0.49; 2-week MD, 0.21; 95% CI, 0.004-0.42; 4-week MD, 0.34; 95% CI, 0.13-0.55) (Table 2).

**Table 2.** Rates of LOC by GFAP and NfL Trajectory Subgroup

Subgroup	Participants, No. (%)		
	LOC	No LOC	Unsure LOC
GFAP^a^			
Extreme and prolonged (n = 12)	11 (92)	0	1 (8)
Moderate and transient (n = 63)	14 (22)	44 (70)	5 (8)
NfL			
Extreme and prolonged (n = 6)	6 (100)	0	0
Moderate and prolonged (n = 12)	8 (67)	2 (17)	2 (17)
Minimal or no change (n = 63)	13 (21)	46 (73)	4 (6)

Abbreviations: GFAP, glial fibrillary acidic protein; LOC, loss of consciousness; NfL, neurofilament light.

^a^ Six participants were not included in the GFAP trajectory subgroup analysis as they were missing a 24-hour blood sample.

### LOC Associations With Biomarkers, Symptoms, Cognition, and RTT

**Figure 3.**
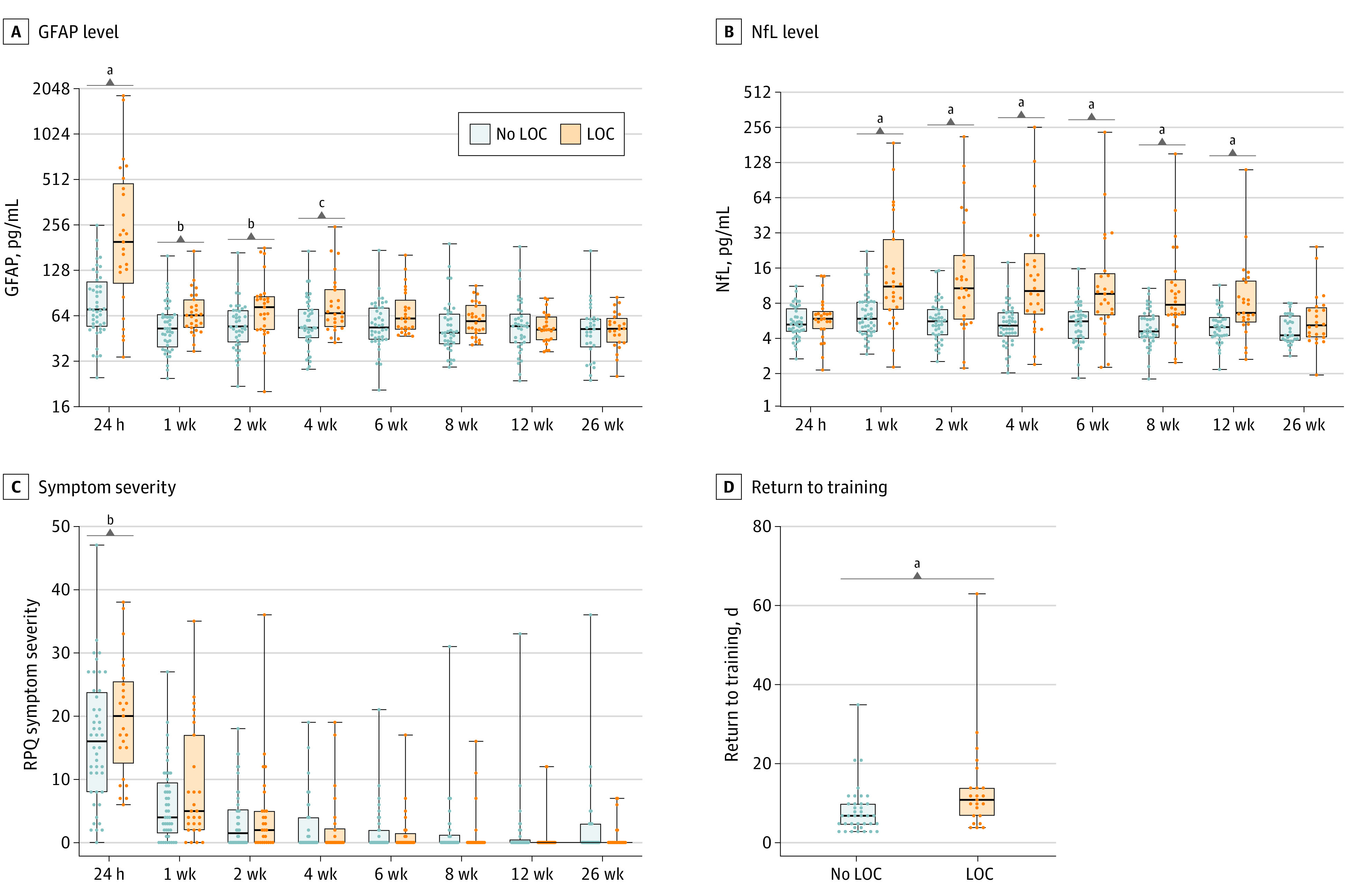
Biomarker and Clinical Recovery in Sport-Related Concussion (SRC) Participants With and Without Loss of Consciousness (LOC) Serum glial fibrillary acidic protein (GFAP) levels were higher at 24 hours, 1 week, 2 weeks, and 4 weeks in SRC participants with LOC compared with those without (A). Similarly for serum neurofilament light (NfL), levels were higher in participants with LOC at 1 week, 2 weeks, 4 weeks, 6 weeks, 8 weeks, and 12 weeks compared with participants without LOC (B). Symptom evaluation with the Rivermead Post Concussion Questionnaire (RPQ) revealed a greater severity of symptoms in SRC participants with vs without LOC at 24 hours, but no other time points (C). SRC participants with LOC reported a greater time to return to training than participants without LOC (D). With return to training affected by end of season, COVID-19 lockdowns, or failure to return to training, 15 cases were not included. Box plots show the minimum, lower quartile, median, upper quartile, and maximum value. ^a^*P* < .001. ^b^*P* < .05. ^c^*P* < .01.

Increased GFAP levels were found from 24 hours to 4 weeks (24-hour MD, Ln pg/mL, 1.01; 95% CI, 0.77-1.24; 1-week MD, 0.27; 95% CI, 0.06-0.49; 2-week MD, 0.21; 95% CI, 0.004-0.42; and 4-week MD, 0.34; 95% CI, 0.13-0.55) in the SRC participants with LOC compared with those without LOC (Figure 3A). Furthermore, NfL levels were increased in SRC participants with LOC from 1 week to 12 weeks compared with those without LOC (1-week MD, 0.73 [95% CI, 0.42-1.03]; 2-week MD, 0.91 [95% CI, 0.61-1.21]; 4-week MD, 0.90 [95% CI, 0.59-1.20]; 6-week MD, 0.81 [95% CI, 0.50-1.13]; 8-week MD, 0.73 [95% CI, 0.42-1.04]; and 12-week MD, 0.54 [95% CI, 0.22-0.85]) (Figure 3B). Individuals with LOC had more 24-hour symptoms compared with those without LOC (Figure 3C). No such differences were found on Cogstate evaluation (eFigure 6 in Supplement 1). Full results are reported in eTable 10 in Supplement 1.

Time to RTT was greater in SRC participants with LOC compared with those without LOC (IRR, 1.65; 95% CI, 1.41-1.93) (Figure 3D; eTable 9 in Supplement 1). Secondary analyses assessed the utility of 24-hour GFAP, 1-week NfL, and LOC to estimate symptom severity at 24 hours and time to RTT. While only LOC was associated with 24-hour symptoms, 24-hour GFAP, 1-week NfL, and LOC were each associated with RTT time; eTable 11 in Supplement 1 provides full results.

Sensitivity analyses were conducted to determine whether having a concussion in the previous 12 months (8 cases) or repeated participation (9 cases) in the study confounded the primary outcomes. No change in statistical modeling of biomarker trajectory subgroups resulted. In longitudinal analyses, omission of these cases resulted in a small number of minor changes in significant time points; however, mean differences, CIs, and primary conclusions remained highly similar. In addition, we included the number of previous concussions, sex, and psychiatric disease as covariates in primary analyses, finding no significant differences in statistical modeling or post hoc comparisons; eTable 12 in Supplement 1 provides full results.

## Discussion

To our knowledge, this study provides the most comprehensive temporal profile of serum GFAP and NfL after SRC to date. At a whole group level, we observed a pronounced increase in GFAP levels at 24 hours and a delayed but prolonged increase in NfL for 12 weeks. Distinct trajectory subgroups emerged for GFAP (extreme prolonged and moderate transient) and NfL (extreme prolonged, moderate prolonged, and minimal or no change). Participants in the extreme GFAP and extreme/moderate NfL subgroups took longer to RTT than the moderate GFAP and minimal or no change NfL subgroups and had higher rates of LOC. Furthermore, stratifying by LOC showed individuals with LOC had greater and more prolonged differences in GFAP and NfL.

Our study adds a unique perspective to previous studies reporting delayed and prolonged NfL increases after mTBI or SRC,^16,32^ by providing more comprehensive temporal profiling (8 time points, narrow time-bins, and low attrition) and revealing that group-level NfL changes are primarily driven by a subset of SRC cases. In this study, only 22% of participants with SRC demonstrated a substantial change in NfL levels (ie, moderate or extreme NfL subgroups), with levels increased above those of control participants for at least 12 weeks; however, the long half-life of NfL makes it difficult to determine the cessation of axonal injury and brain clearance of this protein. To offer insights into this, we performed a secondary analysis on the timing of peak NfL levels in athletes in the moderate or extreme NfL subgroups who completed all time points out to 12 weeks (n = 14 of 18). Five participants had peak NfL levels at 1 week, 5 at 2 weeks, and 4 at 4 weeks. Variable timing of NfL peaks likely indicate interindividual differences in either the duration of axonal damage (potentially involving delayed Wallerian degeneration), the duration of NfL clearance from the brain, or a combination of both. In any case, NfL changes persisting for several weeks post SRC are likely reflective of incomplete neurobiologic recovery. We also found that NfL increases were prognostic of prolonged RTT times, a finding consistent with previous research.^32^ Considering recent preclinical evidence that high NfL levels after mTBI predict increased vulnerability to reinjury,^19^ it appears likely that NfL measures have utility in SRC management.

We observed a prevalent increase in GFAP levels in SRC cases at 24 hours, providing further evidence of its likely diagnostic utility. By defining the profile of GFAP subacutely, we discovered evidence of differences between the overall SRC and control groups at 4 weeks. Deeper analyses revealed that a prolonged GFAP increase was present in a subset of SRC participants, with these individuals also taking longer to RTT. Given its relatively rapid clearance from the blood,^27^ increased GFAP levels likely reflect recent astroglial damage; however, ongoing brain clearance and astrogliosis may contribute.^35,36^ These findings add to the interpretation from the NfL analyses, suggesting that a subset of SRC cases can have neurobiologic alterations that persist for at least 4 weeks, occurring in the absence of persisting symptoms or cognitive impairment.

A notable finding for the extreme GFAP and NfL subgroups was that nearly all participants reported LOC. Previous studies have found more substantial increases in these biomarkers in mTBI or SRC participants with LOC and/or posttraumatic amnesia^24,35^; however, to our knowledge, no studies have investigated this at serial and consistent time points beyond the first week of injury or investigated associations with LOC alone. We found that athletes with LOC had extreme and prolonged increases in both NfL and GFAP levels, with NfL levels increased from 1 week through 12 weeks and GFAP levels increased from 24 hours through 4 weeks. These findings suggest that LOC may be useful to estimate the extent and duration of neurobiologic changes after SRC.

This study adds to evidence indicating value in timely and serial measures of NfL and GFAP after mTBI or SRC.^23,24,32,36^ Although questions remain and challenges must be addressed for this to become clinical practice, immediate insights can be gleaned from our findings. First, we provide more evidence that neurobiologic recovery may extend beyond clinical recovery and that, in some cases, this is likely to be at least 4 weeks. Second, considering that participants with the most pronounced increase in GFAP levels at 24 hours were found to be more likely to have prolonged increases in both biomarkers, acute GFAP alone is likely to have some utility for estimating neurobiologic recovery. Third, and perhaps most immediately translatable, we observed that individuals with LOC were more likely to have significant and persisting brain cell injury. The detectability of LOC by club staff, supporters, video review, or self-reporting presents an opportunity for prompt integration into clinical management at all levels of sport.

### Limitations

This study has limitations. Most participants were male and all were younger adults, preventing insights into biological sex and age. Although our data feature high temporal resolution due to the number of time points and narrow time-bins, our sample size was relatively small for modeling of trajectory subgroups, and power calculations were not performed for this component. As such, additional subgroups, such as a minimal or no change subgroup for GFAP, may be unveiled in larger studies. In addition, 9 individuals participated twice, which may have influenced some findings. While we show evidence that GFAP and NfL levels are comparable between uninjured athletes and those with minor musculoskeletal injury, larger studies are required to confirm that musculoskeletal injuries do not affect biomarker profiles and utility. In addition, subtle decreases in serum GFAP levels have been reported immediately after exercise (but not at 45 minutes post exercise)^37^; however, we did not capture time since exercise. Although sensitivity analyses found that excluding participants with a recent SRC and adding psychiatric disease, number of prior concussions, and sex as covariates did not affect our conclusions, larger studies are required to determine how these factors may influence biomarker profiles. Finally, some outcomes (ie, symptoms, LOC, and RTT) were self-reported; however, most LOC cases were independently corroborated by club medical reports.

## Conclusions

The findings of this cohort study suggest that serial blood measures of GFAP and NfL levels could help identify and track neurobiologic injury and recovery after SRC, complementing the evaluation of clinical recovery in informing return to play decisions. The association of LOC with substantial and prolonged biomarker increases supports the potential adoption of more-conservative return to play timelines in cases where this clinical feature is identified.

## Notes

Supplement 1. **eFigure 1.** Representative Flow Chart of Participant Recruitment, Time-Point Attrition, and Exclusion From Primary Group Analyses **eFigure 2.** The Profiles of GFAP and NfL in Musculoskeletal Injury and Healthy Control Participants **eFigure 3.** The Profile of RPQ Symptom Severity Score in Sport-Related Concussion and Control Participants **eFigure 4.** Cognitive Performance Over Time in Sport-Related Concussion and Control Participants **eFigure 5.** Cognitive Profiles of Sport-Related Concussion Participants Grouped by GFAP and NfL Subgroups **eFigure 6.** Cognitive Profile for Sport-Related Concussion Participants With and Without a Loss of Consciousness **eTable 1.** Sample Size, Median and Interquartile Range for Sport-Related Concussion, Musculoskeletal Control and Healthy Control Participants at Each Time-Point **eTable 2.** Model Fit Statistics for Growth Mixture Modeling to Determine the Number of GFAP Subgroups **eTable 3.** Model Fit Statistics for Growth Mixture Modeling to Determine the Number of NfL Subgroups **eTable 4.** Group Assignments for Individuals Who Participated Twice **eTable 5.** Post Hoc Results for Comparisons of Serum NfL Over Time in the Musculoskeletal Injury Group Compared to Healthy Control Group **eTable 6.** Post Hoc Results for Longitudinal Outcomes in Sport-Related Concussion (SRC) Participants Compared to Control Participants **eTable 7.** Post Hoc Results for Longitudinal Outcomes in Sport-Related Concussion (SRC) Participants Grouped by GFAP Subgroup **eTable 8.** Post Hoc Results for Longitudinal Outcomes in Sport-Related Concussion (SRC) Participants Grouped by NfL Subgroup **eTable 9.** Linear Mixed Models Results for GFAP Subgroup, NfL Subgroup and Loss of Consciousness (LOC) as Predictors of Time to Return to Training **eTable 10.** Post-Hoc Results for Comparisons of Longitudinal Outcomes in Sport-Related Concussion (SRC) Participants With and Without Loss of Consciousness **eTable 11.** Linear Mixed Model Results for 24hr GFAP, 1w NfL and LOC as Predictors of 24hr RPQ Symptom Severity and Time to Return to Training **eTable 12.** Post Hoc Results for Sensitivity Analyses

Supplement 2.
Data Sharing Statement
