# Supplementary material for: Biomarkers of Neurobiologic Recovery in Adults With Sport-Related Concussion
Source: JAMA Netw Open. 2024 Jun 7;7(6):e2415983. doi: 10.1001/jamanetworkopen.2024.15983 (PMC11161851; doi:10.1001/jamanetworkopen.2024.15983)
Supplement: Supplement 1. — eFigure 1. Representative Flow Chart of Participant Recruitment, Time-Point Attrition, and Exclusion From Primary Group Analyses eFigure 2. The Profiles of GFAP and NfL in Musculoskeletal Injury and Healthy Control Participants eFigure 3. The Profile of RPQ Symptom Severity Score in Sport-Related Concussion and Control Participants eFigure 4. Cognitive Performance Over Time in Sport-Related Concussion and Control Participants eFigure 5. Cognitive Profiles of Sport-Related Concussion Participants Grouped by GFAP and NfL Subgroups eFigure 6. Cognitive Profile for Sport-Related Concussion Participants With and Without a Loss of Consciousness eTable 1. Sample Size, Median and Interquartile Range for Sport-Related Concussion, Musculoskeletal Control and Healthy Control Participants at Each Time-Point eTable 2. Model Fit Statistics for Growth Mixture Modeling to Determine the Number of GFAP Subgroups eTable 3. Model Fit Statistics for Growth Mixture Modeling to Determine the Number of NfL Subgroups eTable 4. Group Assignments for Individuals Who Participated Twice eTable 5. Post Hoc Results for Comparisons of Serum NfL Over Time in the Musculoskeletal Injury Group Compared to Healthy Control Group eTable 6. Post Hoc Results for Longitudinal Outcomes in Sport-Related Concussion (SRC) Participants Compared to Control Participants eTable 7. Post Hoc Results for Longitudinal Outcomes in Sport-Related Concussion (SRC) Participants Grouped by GFAP Subgroup eTable 8. Post Hoc Results for Longitudinal Outcomes in Sport-Related Concussion (SRC) Participants Grouped by NfL Subgroup eTable 9. Linear Mixed Models Results for GFAP Subgroup, NfL Subgroup and Loss of Consciousness (LOC) as Predictors of Time to Return to Training eTable 10. Post-Hoc Results for Comparisons of Longitudinal Outcomes in Sport-Related Concussion (SRC) Participants With and Without Loss of Consciousness eTable 11. Linear Mixed Model Results for 24hr GFAP, 1w NfL and LOC as Predictors of 24hr RPQ Symptom Seve [file jamanetwopen-e2415983-s001.pdf]

Supplementary Online Content

O'Brien WT, Spitz G, Xie B, et al. Neurobiologic recovery in adults with sport-related concussion. *JAMA Netw Open*. 2024;7(6):e2415983. doi:10.1001/jamanetworkopen.2024.15983

eFigure 1. Representative Flow Chart of participant Recruitment, Time-Point Attrition, and Exclusion From Primary Group Analyses
eFigure 2. The Profiles of GFAP and NfL in Musculoskeletal Injury and Healthy Control Participants
eFigure 3. The Profile of RPQ Symptom Severity Score in Sport-Related Concussion and Control Participants
eFigure 4. Cognitive Performance Over Time in Sport-Related Concussion and Control Participants
eFigure 5. Cognitive Profiles of Sport-Related Concussion Participants Grouped by GFAP and NfL Subgroups
eFigure 6. Cognitive Profile for Sport-Related Concussion Participants With and Without a Loss of Consciousness
eTable 1. Sample Size, Median and Interquartile Range for Sport-Related Concussion, Musculoskeletal Control and Healthy Control Participants at Each Time-Point
eTable 2. Model Fit Statistics for Growth Mixture Modeling to Determine the Number of GFAP Subgroups
eTable 3. Model Fit Statistics for Growth Mixture Modeling to Determine the Number of NfL Subgroups
eTable 4. Group Assignments for Individuals Who Participated Twice
eTable 5. Post Hoc Results for Comparisons of Serum NfL Over Time in the Musculoskeletal Injury Group Compared to Healthy Control Group
eTable 6. Post Hoc Results for Longitudinal Outcomes in Sport-Related Concussion (SRC) Participants Compared to Control Participants
eTable 7. Post Hoc Results for Longitudinal Outcomes in Sport-Related Concussion (SRC) Participants Grouped by GFAP Subgroup
eTable 8. Post Hoc Results for Longitudinal Outcomes in Sport-Related Concussion (SRC) Participants Grouped by NfL Subgroup
eTable 9. Linear Mixed Models Results for GFAP Subgroup, NfL Subgroup and Loss of Consciousness (LOC) as Predictors of Time to Return to Training
eTable 10. Post-Hoc Results for Comparisons of Longitudinal Outcomes in Sport-Related Concussion (SRC) Participants With and Without Loss of Consciousness
eTable 11. Linear Mixed Model Results for 24hr GFAP, 1w NfL and LOC as Predictors of 24hr RPQ Symptom Severity and Time to Return to Training
eTable 12. Post Hoc Results for Sensitivity Analyses

This supplementary material has been provided by the authors to give readers additional information about their work.

© 2024 O'Brien WT et al. *JAMA Network Open.*

eFigure 1: Representative flow chart of participant recruitment, time-point attrition, and exclusion from primary group analyses.

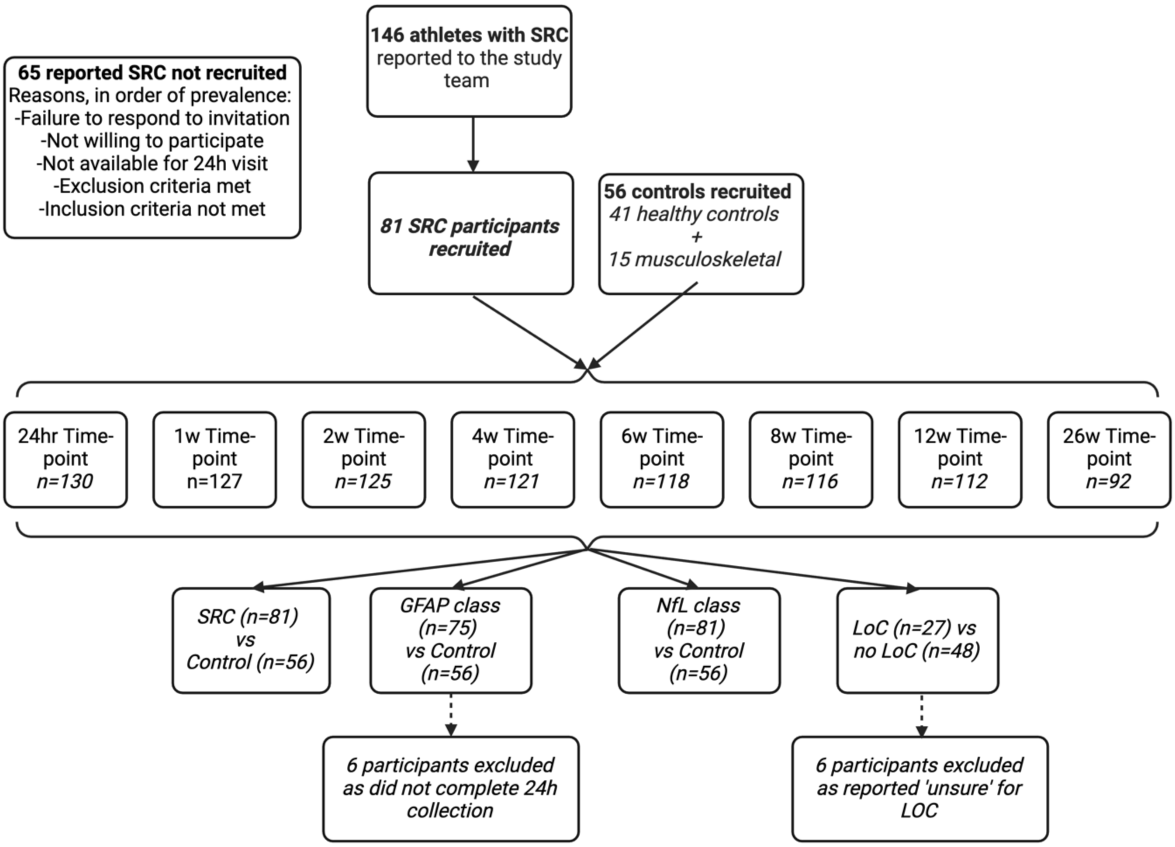

SRC: Sport-Related Concussion. GFAP: Glial Fibrillary Acidic Protein. NfL: Neurofilament light. LOC: Loss of Consciousness. Created with BioRender.com

© 2024 O'Brien WT et al. *JAMA Network Open.*

eFigure 2: The profiles of GFAP and NfL in musculoskeletal injury and healthy control participants.

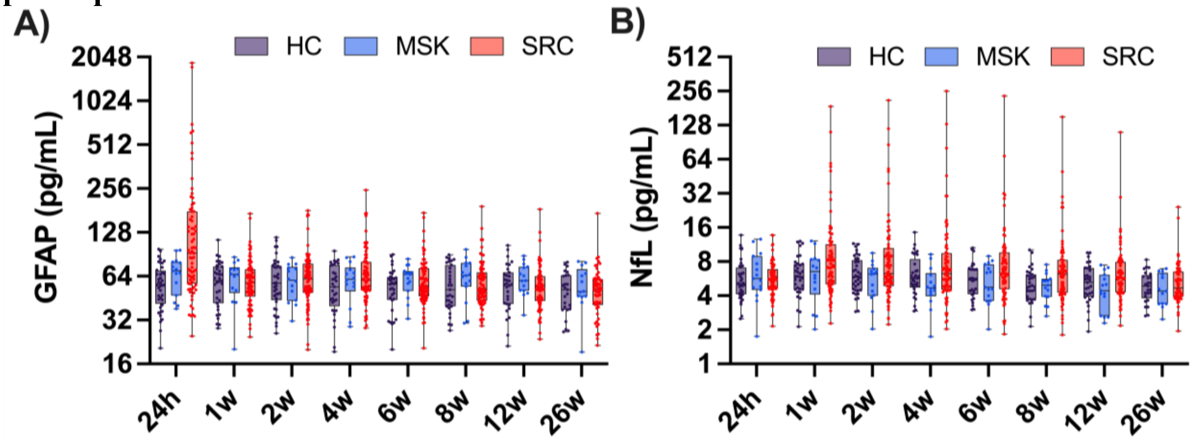

No differences were found in the profiles of GFAP (**A**) or NfL (**B**) in musculoskeletal injury or healthy control participants. Statistical comparisons are made between the MSK and healthy control groups only. *GFAP: Glial Fibrillary Acidic Protein. NfL: Neurofilament light. MSK: Musculoskeletal. HC: Healthy Control. SRC: Sport-related concussion.*

© 2024 O'Brien WT et al. *JAMA Network Open.*

eFigure 3: The profile of RPQ symptom severity score in sport-related concussion and control participants.

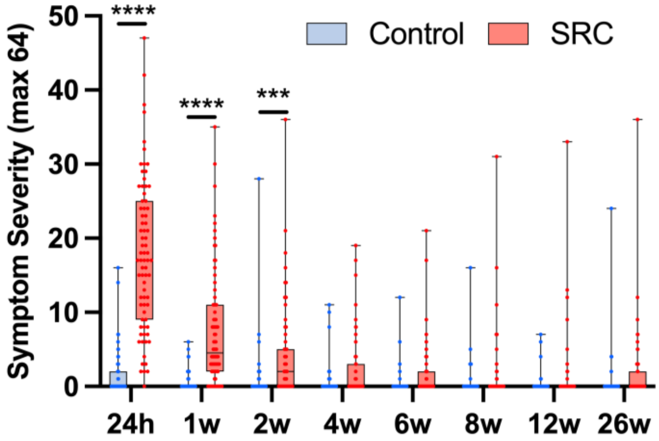

Symptom evaluations performed with the symptom severity score of the Rivermead Post Concussion Questionnaire revealed a greater severity of symptoms in the sport-related concussion (SRC) participants compared to control participants at 24hrs ($p<0.0001$), 1w ($p<0.0001$) and 2w ($p=0.0009$). **** and **** denotes $p<0.001$ and $p<0.0001$ respectively. SRC: Sport-related concussion.*

© 2024 O'Brien WT et al. *JAMA Network Open.*

eFigure 4: Cognitive performance over time in sport-related concussion and control participants.

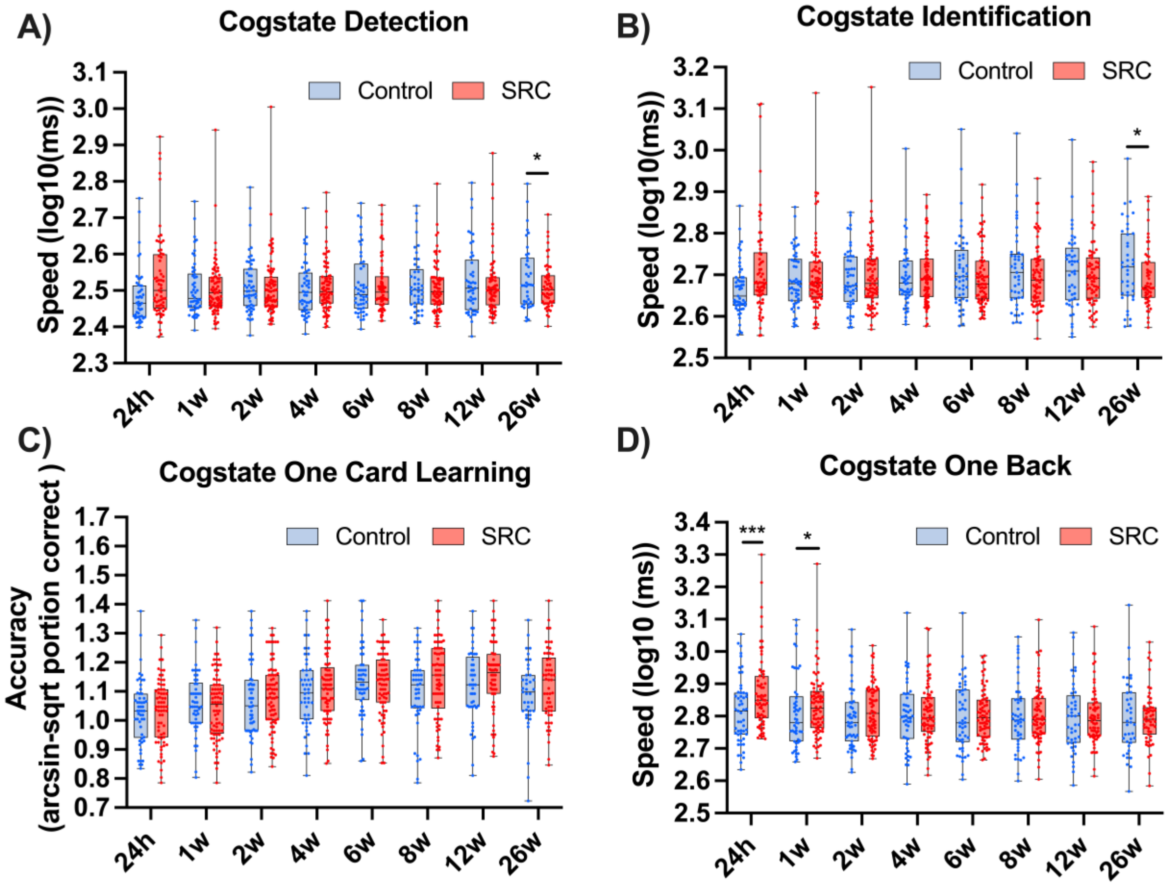

Cognitive assessments performed with the Cogstate found an increased speed in the SRC participants compared to control participants in the Detection (**A**; p=0.036) and Identification task (**B**; p=0.022). While no differences were found in the One Card Learning (**C**), decreased speed in the One Back task was found at 24h (**D**; p=0.0009) and 1w (p=0.03), * *and* ****denotes p<0.05 and 0.001 respectively.*

© 2024 O'Brien WT et al. *JAMA Network Open.*

eFigure 5: Cognitive profiles of sport-related concussion participants grouped by GFAP and NfL subgroup.

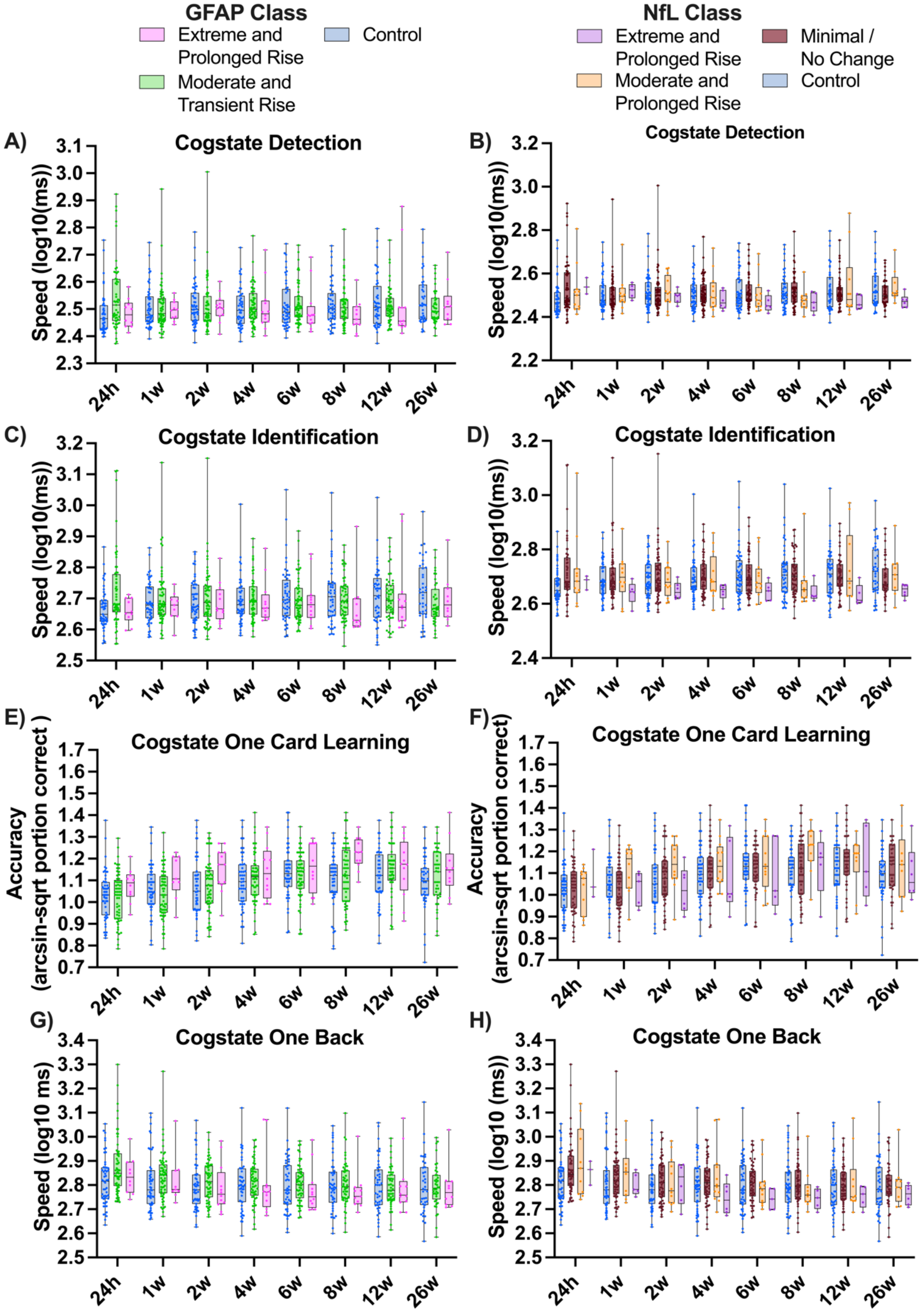

Cognitive assessment with the Cogstate revealed no differences in sport-related concussion (SRC) in GFAP or NfL subgroups compared to control participants for the Detection (**A, B**), Identification (**C, D**), One Card Learning (**E, F**) or One Back task (**G, H**). *Data is presented as min-max box plots. GFAP: Glial fibrillary acidic protein. NfL: Neurofilament light.*

© 2024 O'Brien WT et al. *JAMA Network Open.*

eFigure 6: Cognitive profile for sport-related concussion participants with and without a loss of consciousness.

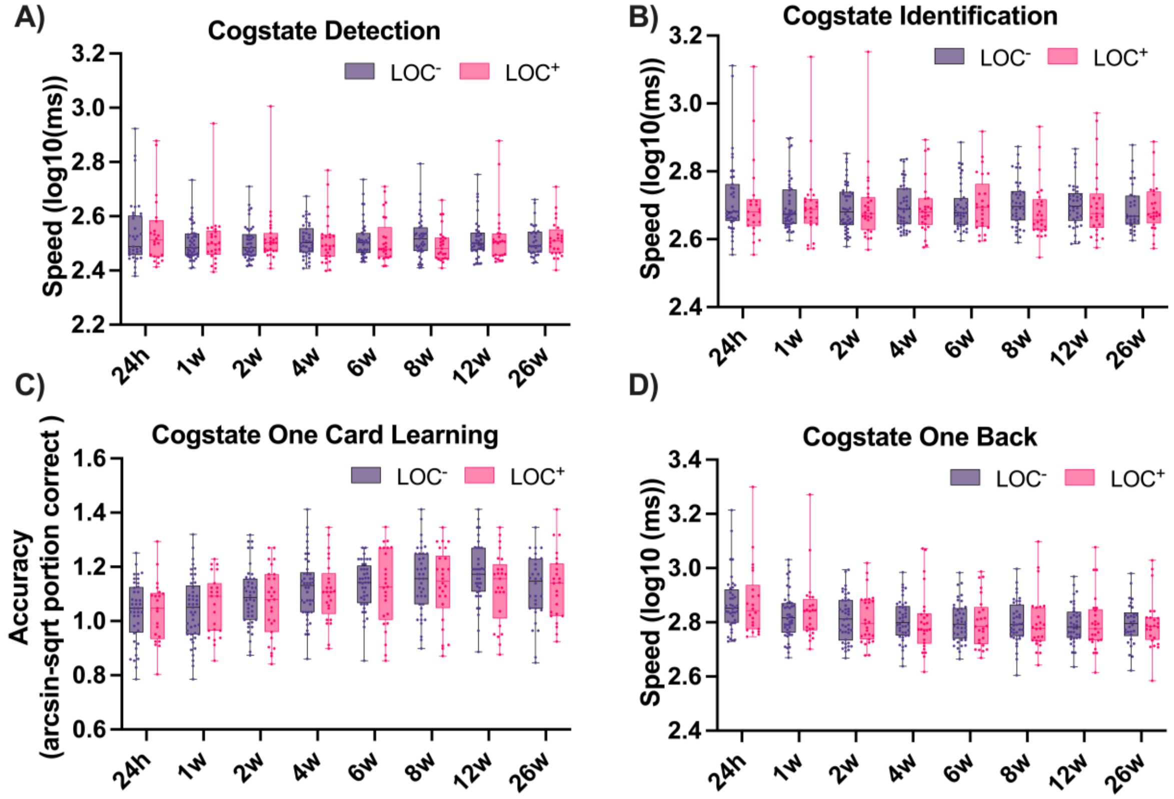

Cognitive assessments performed with the Cogstate found no differences in the Detection (**C**), Identification (**D**), One Card Learning (**E**) or the One Back task (**F**) between sport-related concussion participants with and without loss of consciousness (LOC). *sLOC*$^-$: No self-reported loss of consciousness. sLOC$^+$: Self-reported loss of consciousness.

© 2024 O'Brien WT et al. *JAMA Network Open.*

eTable 1: Sample size, median and interquartile range for sport-related concussion, musculoskeletal injury and healthy control groups at each time-point.

	Sport-related concussion			Musculoskeletal			Healthy Controls			Overall		
**Collection**	**n**	**Median**	**IQR**	**n**	**Median**	**IQR**	**n**	**Median**	**IQR**	**n**	**Median**	**IQR**
**0w (h)**	75	25.5	22.8-31.2	15	25.5	24.8-42.2	40	22.9	20.1-25.9	130	25.0	22.3-28.8
**1w (d)**	78	8.1	7.9-8.9	14	8.0	7.9-9.1	35	8.0	7.8-8.2	127	8.0	7.9-8.9
**2w (d)**	74	15.1	14.9-16.0	14	15.7	15.0-16.7	37	15.0	14.8-15.2	125	15.1	14.9-15.9
**4w (d)**	74	29.1	28.9-30.0	14	29.5	29.0-30.2	33	29.0	28.9-30.1	121	29.1	28.9-30.1
**6w (d)**	71	43.1	42.9-44.1	15	44.2	44.0-45.6	32	43.0	42.8-44.2	118	43.1	42.9-44.2
**8w (d)**	70	57.1	56.9-58.1	15	58.0	57.0-58.5	31	56.9	56.8-57.1	116	57.1	56.9-58.2
**12w (d)**	66	85.2	84.9-86.1	15	85.1	85.0-86.2	31	85.0	84.9-86.0	112	85.1	84.9-86.2
**26w (d)**	54	185.0	183.0-197.9	13	190.2	184.2-205.0	25	184.0	182.9-186.0	92	184.9	183.0-197.1

n: number. IQR: Interquartile range. h: hours. d: days

© 2024 O'Brien WT et al. *JAMA Network Open.*

eTable 2: Model fit statistics for growth mixture modeling to determine the number of GFAP subgroups.

Time polynomial	Slope	Intercept	No. of Classes	AIC	BIC	loglik	%class1	%class2	%class3	%class4
1	Fixed	Fixed	1	817.32	824.28	-405.66	100			
1	Fixed	Fixed	2	701.06	714.97	-344.53	26.67	73.33		
1	Fixed	Fixed	3	651.39	672.25	-316.69	5.33	58.66	36	
1	Fixed	Fixed	4	630.05	657.86	-303.02	25.33	53.33	5.33	16
1	Fixed	Random	1	644.95	654.22	-318.47	100			
1	Fixed	Random	2	627.51	646.05	-305.75	9.33	90.67		
1	Fixed	Random	3	630.15	657.96	-303.08	89.33	9.33	1.33	
1	Fixed	Random	4	632.23	669.31	-300.11	10.67	48	37.33	4
1	Random	Random	1	633.84	647.75	-310.92	100			
1	Random	Random	2	632.66	655.83	-306.33	5.33	94.67		
1	Random	Random	3	631.30	663.75	-301.65	90.67	8	1.33	
1	Random	Random	4	638.90	680.62	-301.45	1.33	1.33	89.33	8
2	Fixed	Fixed	1	800.96	810.23	-396.48	100			
2	Fixed	Fixed	2	669.90	690.76	-325.95	74.67	25.33		
2	Fixed	Fixed	3	611.05	643.49	-291.52	36	5.33	58.67	
2	Fixed	Fixed	4	584.38	628.41	-273.19	18.67	50.67	5.33	25.33
2	Fixed	Random	1	609.51	621.09	-299.75	100			
2	Fixed	Random	2	552.52	575.69	-266.26	10.67	89.33		
2	Fixed	Random	3	558.73	593.49	-264.37	10.67	86.67	2.67	
2	Fixed	Random	4	563.52	609.87	-261.76	1.33	10.67	69.33	18.67

© 2024 O'Brien WT et al. *JAMA Network Open.*

eTable 2 (continued) Model fit statistics for growth mixture modelling to determine the number of GFAP subgroups.

Time polynomial	Slope	Intercept	No. of Classes	AIC	BIC	loglik	%class1	%class2	%class3	%class4
2	Random	Random	1	571.85	595.03	-275.93	100			
2	Random	Random	2	558.94	593.70	-264.47	10.67	89.33		
2	Random	Random	3	559.74	606.09	-259.87	88	10.67	1.33	
2	Random	Random	4	565.27	623.21	-257.64	1.33	10.67	13.33	74.67
3	Fixed	Fixed	1	792.09	803.68	-391.05	100			
3	Fixed	Fixed	2	650.84	676.33	-314.42	76	24		
3	Fixed	Fixed	3	589.64	629.04	-277.82	58.67	5.333	36	
3	Fixed	Fixed	4	556.99	610.29	-255.49	50.67	5.33	18.67	25.33
3	Fixed	Random	1	589.65	603.56	-288.83	100			
**3**	**Fixed**	**Random**	**2**	**507.65**	**535.46**	**-241.82**	**16**	**84**		
3	Fixed	Random	3	517.15	558.86	-240.57	53.33	32	14.67	
3	Fixed	Random	4	506.44	562.06	-229.22	4	17.33	77.33	1.33
3	Random	Random	1	532.52	567.29	-251.26	100			
3	Random	Random	2	519.32	567.99	-238.66	84	16		
3	Random	Random	3	534.88	597.45	-240.44	65.33	16	18.67	
3	Random	Random	4	533.58	610.06	-233.79	18.67	20	38.67	22.67

The chosen model is bolded. AIC: Akaike Information Criterion. BIC: Bayesian Information Criterion.

© 2024 O'Brien WT et al. *JAMA Network Open.*

eTable 3 Model fit statistics for growth mixture modeling to determine the number of NfL subgroups.

Time polynomial	Slope	Intercept	No. of Classes	AIC	BIC	loglik	%class1	%class2	%class3	%class4
1	Fixed	Fixed	1	1207.03	1214.21	-600.51	100			
1	Fixed	Fixed	2	854.38	868.74	-421.19	92.59	7.41		
1	Fixed	Fixed	3	705.98	727.52	-343.98	39.51	53.09	7.41	
1	Fixed	Fixed	4	621.21	649.94	-298.60	53.09	2.47	39.51	4.94
1	Fixed	Random	1	646.09	655.67	-319.04	100			
1	Fixed	Random	2	556.02	575.17	-270.01	8.64	91.36		
1	Fixed	Random	3	551.77	580.50	-263.89	14.81	7.41	77.78	
1	Fixed	Random	4	555.95	594.26	-261.98	7.41	17.28	27.16	48.15
1	Random	Random	1	593.20	607.56	-290.60	100			
1	Random	Random	2							
1	Random	Random	3	555.34	588.86	-263.67	12.35	7.41	80.25	
1	Random	Random	4	559.80	602.90	-261.90	27.16	17.28	48.15	7.41
2	Fixed	Fixed	1	1208.11	1217.69	-600.05	100			
2	Fixed	Fixed	2	852.91	874.46	-417.45	92.59	7.41		
2	Fixed	Fixed	3	702.19	735.71	-337.09	7.41	38.27	54.32	
2	Fixed	Fixed	4	661.38	706.87	-311.69	7.41	40.74	34.57	17.28
2	Fixed	Random	1	641.76	653.73	-315.88	100			
2	Fixed	Random	2	519.79	543.74	-249.90	85.19	14.81		
2	Fixed	Random	3	504.54	540.46	-237.27	7.41	12.35	80.25	
2	Fixed	Random	4	508.88	556.77	-234.44	37.04	7.41	14.814	40.74
2	Random	Random	1	546.26	570.21	-263.13	100			
2	Random	Random	2	497.56	533.48	-233.78	14.81	85.19		
2	Random	Random	3	503.90	551.78	-231.95	16.05	49.38	34.57	
2	Random	Random	4	500.22	560.08	-225.11	35.80	14.81	40.74	8.64

© 2024 O’Brien WT et al. *JAMA Network Open.*

eTable 3 (continued): Model fit statistics for growth mixture modelling to determine the number of NfL subgroups.

Time polynomial	Slope	Intercept	No. of Classes	AIC	BIC	loglik	%class1	%class2	%class3	%class4
3	Fixed	Fixed	1	1205.19	1217.16	-597.60	100			
3	Fixed	Fixed	2	829.45	855.79	-403.73	92.59	7.41		
3	Fixed	Fixed	3	656.81	697.51	-311.40	37.04	7.41	55.56	
3	Fixed	Fixed	4	550.02	605.10	-252.01	2.47	4.94	37.04	55.56
3	Fixed	Random	1	628.55	642.91	-308.27	100			
3	Fixed	Random	2	449.67	478.40	-212.83	82.72	17.28		
**3**	**Fixed**	**Random**	**3**	**411.75**	**454.85**	**-187.88**	**14.81**	**7.41**	**77.78**	
3	Fixed	Random	4	414.02	471.49	-183.01	7.41	38.27	14.81	39.51
3	Random	Random	1	453.65	489.57	-211.82	100			
3	Random	Random	2	405.22	455.51	-181.61	18.52	81.48		
3	Random	Random	3	414.35	479.00	-180.18	45.68	17.28	37.04	
3	Random	Random	4	419.05	498.07	-176.53	28.40	30.86	14.81	25.93

The chosen model is bolded. AIC: Akaike Information Criterion. BIC: Bayesian Information Criterion.

© 2024 O’Brien WT et al. *JAMA Network Open.*

eTable 4: Group Assignments for individuals who participated twice.

Player	Sex	First Group	Second Group
1	Male	SRC (2021)	SRC (2022)
2	Male	SRC (2021)	MSK (2022)
3	Male	MSK (2021)	HC (2022)
4	Male	MSK (2021)	HC (2022)
5	Male	SRC (2021)	HC (2022)
6	Male	HC (2021)	SRC (2022)
7	Male	HC (2022)	SRC (2022)
8	Male	HC (2022)	SRC (2022)
9	Male	SRC (2022)	SRC (2022)

Year recruited denoted by (year).

© 2024 O'Brien WT et al. *JAMA Network Open.*

eTable 5: Post hoc results for comparisons of serum NfL over time in the musculoskeletal injury group compared to healthy control group.

	**24h**	**1w**	**2w**	**4w**	**6w**	**8w**	**12w**	**26w**
**Serum GFAP – *No significant interaction***								
**Serum NfL**								
MD	-0.02	0.002	0.02	0.05	0.08	0.10	0.13	0.08
95% CI	(-0.26, 0.22)	(-0.23, 0.23)	(-0.21, 0.25)	(-0.17, 0.27)	(-0.13, 0.29)	(-0.11, 0.31)	(-0.07, 0.33)	(-0.12, 0.28)
p-value	0.89	0.99	0.86	0.63	0.46	0.33	0.20	0.44

Values are relative to the healthy control group. MD: Mean difference. GFAP: Glial fibrillary acidic protein. NfL: Neurofilament light.

© 2024 O'Brien WT et al. *JAMA Network Open.*

eTable 6: Post-hoc results for longitudinal outcomes in sport-related concussion (SRC) participants compared to control participants.

	**24h**	**1w**	**2w**	**4w**	**6w**	**8w**	**12w**	**26w**
**RPQ Symptom Score**								
MD	16.15	6.78	2.90	1.18	0.67	0.54	0.85	1.13
95% CI	(14.21, 18.09)	(4.98, 8.58)	(1.21, 4.59)	(-0.58, 2.93)	(-1.10, 2.45)	(-1.2, 2.32)	(-0.89, 2.58)	(-0.87, 3.14)
p-value	<.0001	<.0001	0.0009	0.18	0.46	0.55	0.34	0.26
**Cogstate Detection Task (Latency)**								
MD	0.01	0.009	0.007	0.004	-0.0003	-0.004	-0.01	-0.04
95% CI	(-0.01, 0.04)	(-0.02, 0.03)	(-0.02, 0.03)	(-0.02, 0.03)	(-0.02, 0.02)	(-0.03, 0.02)	(-0.04, 0.01)	(-0.07, -0.005)
p-value	0.38	0.46	0.55	0.77	0.98	0.73	0.35	0.03
**Cogstate Identification Task (Latency)**								
MD	0.03	0.02	0.02	0.02	0.02	0.01	0.009	-0.01
95% CI	(-0.004, 0.06)	(-0.005, 0.05)	(-0.007, 0.05)	(-0.009, 0.05)	(-0.01, 0.05)	(-0.01, 0.04)	(-0.02, 0.04)	(-0.05, 0.02)
p-value	0.09	0.11	0.13	0.18	0.24	0.33	0.56	0.55
**Cogstate One Card Learning Task (Accuracy) – No significant interaction**								
**Cogstate One Back Task (Latency)**								
MD	0.06	0.03	0.02	0.004	0.004	0.008	0.004	0.0007
95% CI	(0.023, 0.09)	(0.003, 0.06)	(-0.01, 0.05)	(-0.03, 0.03)	(-0.03, 0.03)	(-0.02, 0.04)	(-0.03, 0.04)	(-0.03, 0.04)
p-value	0.0009	0.03	0.27	0.81	0.82	0.64	0.81	0.97
**Serum GFAP**								
MD	0.66	0.03	-0.003	0.17	0.04	-0.005	-0.07	-0.03
95% CI	(0.50, 0.82)	(-0.12, 0.19)	(-0.15, 0.15)	(0.02, 0.32)	(-0.11, 0.19)	(-0.15, 0.14)	(-0.21, 0.07)	(-0.18, 0.12)
p-value	<.0001	0.66	0.97	0.03	0.60	0.94	0.32	0.68
**Serum NfL**								
MD	0.07	0.31	0.38	0.31	0.27	0.36	0.25	0.03
95% CI	(-0.14, 0.27)	(0.12, 0.51)	(0.19, 0.58)	(0.12, 0.51)	(0.07, 0.47)	(0.15, 0.56)	(0.04, 0.46)	(-0.18, 0.25)
p-value	0.52	0.002	0.0002	0.002	0.007	0.0009	0.02	0.75

Values are relative to the control group. MD: Mean difference. CI: Confidence Interval. RPQ: Rivermead Post Concussion Questionnaire. GFAP: Glial fibrillary acidic protein. NfL: Neurofilament light.

© 2024 O'Brien WT et al. *JAMA Network Open.*

eTable 7: Post-hoc results for longitudinal outcomes in sport-related concussion (SRC) participants grouped by GFAP subgroup.

		24h	1w	2w	4w	6w	8w	12w	26w
**RPQ Symptom Score**									
Control – Extreme GFAP	*MD*	-19.74	-9.06	-3.95	-1.90	-2.23	-0.95	-1.04	-1.17
	95% CI	(-24.05, -15.42)	(-12.84, -5.28)	(-7.70, -0.20)	(-5.54, 1.74)	(-5.68, 1.23)	(-4.7, 2.82)	(-4.75, 2.678)	(-5.40, 3.08)
	p-value	<.0001	<.0001	0.02	0.44	0.23	0.71	0.75	0.76
Control – Moderate GFAP	*MD*	-15.42	-6.85	-2.70	-0.94	-1.20	-0.35	-1.14	-1.42
	95% CI	(-17.90, -12.94)	(-9.04, -4.66)	(-4.88, -0.52)	(-3.08, 1.20)	(-3.24, 0.83)	(-2.62, 1.92)	(-3.42, 1.15)	(-4.09, 1.25)
	p-value	<.0001	<.0001	0.01	0.44	0.23	0.71	0.70	0.60
**Cogstate Detection Task (Latency)**									
Control – Extreme GFAP	*MD*	0.005	0.006	0.007	0.009	0.01	0.01	0.02	0.03
	95% CI	(-0.052, 0.06)	(-0.05, 0.06)	(-0.05, 0.06)	(-0.04, 0.06)	(-0.04, 0.06)	(-0.04, 0.07)	(-0.04, 0.07)	(-0.04, 0.11)
	p-value	0.83	0.79	0.75	0.68	0.90	0.79	0.62	0.38
Control – Moderate GFAP	*MD*	-0.01	-0.01	-0.010	-0.005	-0.0009	0.003	0.01	0.04
	95% CI	(-0.05, 0.02)	(-0.04, 0.02)	(-0.04, 0.02)	(-0.04, 0.03)	(-0.03, 0.03)	(-0.03, 0.03)	(-0.02, 0.04)	(-0.002, 0.09)
	p-value	0.62	0.65	0.70	0.68	0.94	0.79	0.62	0.07
**Cogstate Identification Task (Latency)**									
Control – Extreme GFAP	*MD*	-0.005	-0.004	-0.003	-0.0002	0.002	0.005	0.01	0.03
	95% CI	(-0.06, 0.05)	(-0.06, 0.05)	(-0.06, 0.05)	(-0.05, 0.05)	(-0.05, 0.05)	(-0.05, 0.06)	(-0.04, 0.06)	(-0.05, 0.10)
	p-value	0.81	0.85	0.90	0.99	0.91	0.88	0.95	0.53
Control – Moderate GFAP	*MD*	-0.02	-0.02	-0.02	-0.01	-0.007	-0.002	0.009	0.05
	95% CI	(-0.05, 0.008)	(-0.05, 0.01)	(-0.05, 0.01)	(-0.04, 0.02)	(-0.04, 0.02)	(-0.03, 0.03)	(-0.02, 0.04)	(0.0002, 0.09)
	p-value	0.22	0.33	0.48	0.85	0.91	0.88	0.95	0.05
**Cogstate One Card Learning Task (Accuracy)**									
Control – Extreme GFAP	*MD*	-0.06	-0.06	-0.06	-0.06	-0.06	-0.06	-0.06	-0.05
	95% CI	(-0.14, 0.02)	(-0.14, 0.02)	(-0.13, 0.02)	(-0.13, 0.01)	(-0.13, 0.01)	(-0.14, 0.02)	(-0.14, 0.02)	(-0.15, 0.05)
	p-value	0.13	0.10	0.09	0.09	0.14	0.17	0.22	0.52
Control – Moderate GFAP	*MD*	0.01	0.008	0.004	-0.003	-0.009	-0.01	-0.02	-0.01
	95% CI	(-0.03, 0.06)	(-0.04, 0.05)	(-0.04, 0.05)	(-0.05, 0.04)	(-0.05, 0.03)	(-0.06, 0.03)	(-0.07, 0.03)	(-0.07, 0.05)
	p-value	0.53	0.67	0.82	0.86	0.61	0.45	0.30	0.60

© 2024 O’Brien WT et al. *JAMA Network Open.*

eTable 7 (continued): Post-hoc results for longitudinal outcomes in sport-related concussion (SRC) participants grouped by GFAP subgroup.

		24h	1w	2w	4w	6w	8w	12w	26w
**Cogstate One Back Task (Latency) –** ***No significant interaction***									
**Serum GFAP**									
Control – Extreme GFAP	*MD*	-2.20	-0.27	-0.39	-0.48	-0.29	-0.10	0.04	0.14
	95% CI	(-2.50, -1.89	(-0.57, 0.03)	(-0.70, -0.09)	(-0.78, -0.17)	(-0.59, 0.02)	(-0.40, 0.21)	(-0.26, 0.35)	(-0.17, 0.45)
	p-value	<.0001	0.049	0.003	0.0006	0.046	0.65	0.82	0.47
Control – Moderate GFAP	*MD*	-0.38	0.03	0.03	-0.10	-0.02	0.01	0.07	0.01
	95% CI	(-0.55, -	(-0.14, 0.21)	(-0.15, 0.20)	(-0.28, 0.07)	(-0.19, 0.16)	(-0.16, 0.19)	(-0.11, 0.25)	(-0.18, 0.20)
	p-value	<.0001	0.68	0.73	0.17	0.81	0.85	0.82	0.89

MD: Mean difference. CI: Confidence Interval. RPQ: Rivermead Post Concussion Questionnaire. GFAP: Glial fibrillary acidic protein.

© 2024 O'Brien WT et al. *JAMA Network Open.*

eTable 8: Post hoc results for longitudinal outcomes in sport-related concussion (SRC) participants grouped by NfL subgroup.

		24h	1w	2w	4w	6w	8w	12w	26w
	**RPQ Symptom Score**								
Control – Extreme NfL	*MD*	-17.95	-10.01	-5.21	-1.51	-1.36	-1.66	0.22	0.72
	95% CI	(-24.94, -	(-15.57, -4.44)	(-10.71, 0.28)	(-6.87, 3.86)	(-6.53, 3.81)	(-7.44, 4.12)	(-5.50, 5.93)	(-4.93, 6.37)
	p-value	<.0001	<.0001	0.04	0.98	0.94	0.94	0.95	0.85
Control – Moderate NfL	*MD*	-20.33	-8.15	-2.61	-1.00	-1.64	-0.009	-1.04	-1.20
	95% CI	(-25.06, -	(-12.29, -4.01)	(-6.72, 1.49)	(-4.99, 2.98)	(-5.42, 2.14)	(-4.20, 4.18)	(-5.30, 3.23)	(-5.98, 3.57)
	p-value	<.0001	<.0001	0.18	0.98	0.75	1.00	0.93	0.75
Control – Minimal NfL	*MD*	-15.10	-6.80	-2.74	-0.96	-1.21	-0.43	-1.13	-1.54
	95% CI	(-17.85, -	(-9.21, -4.39)	(-5.15, -0.34)	(-3.32, 1.40)	(-3.44, 1.03)	(-2.91, 2.05)	(-3.61, 1.35)	(-4.43, 1.34)
	p-value	<.0001	<.0001	0.02	0.98	0.75	0.94	0.93	0.75
	**Cogstate Detection Task (Latency)**								
Control – Extreme NfL	*MD*	0.011	0.014	0.017	0.02	0.03	0.03	0.04	0.08
	95% CI	(-0.072,	(-0.07, 0.10)	(-0.06, 0.01)	(-0.06, 0.10)	(-0.05, 0.11)	(-0.04, 0.11)	(-0.03, 0.12)	(-0.02, 0.19)
	p-value	0.87	0.78	0.70	0.70	0.68	0.52	0.39	0.12
Control – Moderate NfL	*MD*	-0.02	-0.02	-0.02	-0.01	-0.01	-0.01	-0.008	0.002
	95% CI	(-0.08, 0.04)	(-0.08, 0.04)	(-0.08, 0.04)	(-0.07, 0.04)	(-0.07, 0.05)	(-0.07, 0.06)	(-0.07, 0.05)	(-0.08, 0.08)
	p-value	0.69	0.71	0.70	0.70	0.78	0.73	0.71	0.95
Control – Minimal NfL	*MD*	-0.02	-0.01	-0.01	-0.007	-0.003	0.001	0.009	0.04
	95% CI	(-0.05, 0.02)	(-0.05, 0.02)	(-0.05, 0.02)	(-0.04, 0.03)	(-0.04, 0.03)	(-0.03, 0.03)	(-0.03, 0.04)	(-0.01, 0.09)
	p-value	0.69	0.71	0.70	0.70	0.81	0.93	0.58	0.12

© 2024 O'Brien WT et al. *JAMA Network Open.*

eTable 8 (continued): Post-hoc results for longitudinal outcomes in sport-related concussion (SRC) participants grouped by NfL subgroup.

		**24h**	**1w**	**2w**	**4w**	**6w**	**8w**	**12w**	**26w**
**Cogstate Identification Task (Latency)**									
Control – Extreme NfL	*MD*	0.04	0.04	0.04	0.05	0.05	0.05	0.06	0.10
	95% CI	(-0.04, 0.12)	(-0.03, 0.12)	(-0.042, 0.12)	(-0.03, 0.12)	(-0.03, 0.13)	(-0.02, 0.13)	(-0.02, 0.14)	(-0.002, 0.18)
	p-value	0.32	0.28	0.24	0.24	0.17	0.13	0.12	0.03
Control – Moderate NfL	*MD*	-0.02	-0.02	-0.02	-0.02	-0.01	-0.01	-0.004	0.01
	95% CI	(-0.08, 0.04)	(-0.08, 0.04)	(-0.08, 0.04)	(-0.07, 0.04)	(-0.07, 0.04)	(-0.07, 0.05)	(-0.06, 0.05)	(-0.06, 0.09)
	p-value	0.41	0.44	0.48	0.56	0.66	0.80	0.84	0.58
Control – Minimal NfL	*MD*	-0.02	-0.02	-0.02	-0.01	-0.008	-0.003	0.007	0.04
	95% CI		(-0.05, 0.01)	(-0.05, 0.02)	(-0.05, 0.02)	(-0.04, 0.02)	(-0.04, 0.03)	(-0.03, 0.041)	(0.0001, 0.08)
	p-value	0.19	0.20	0.24	0.43	0.66	0.80	0.71	0.03
**Cogstate One Card Learning Task (Accuracy) – *No significant interaction***									
**Cogstate One Back Task (Latency) – *No significant interaction***									
**Serum NfL**									
Control – Extreme NfL	*MD*	-0.68	-2.40	-2.72	-2.46	-2.48	-2.23	-1.73	-0.84
	95% CI	(-1.15, -0.21)	(-2.82, -1.98)	(-3.14, -2.31)	(-2.89, -2.04)	(-2.93, -2.03)	(-2.69, -1.78)	(-2.18, -1.27)	(-1.28, -0.40)
	p-value	0.0004	<.0001	<.0001	<.0001	<.0001	<.0001	<.0001	<.0001
Control – Moderate NfL		-0.082	-0.82	-0.96	-0.84	-0.87	-0.86	-0.59	-0.02
	95% CI	(-0.41, 0.25)	(-1.13, -0.50)	(-1.27, -0.65)	(-1.16, -0.52)	(-1.20, -0.55)	(-1.19, -0.52)	(-0.93, -0.25)	(-0.37, 0.32)
	p-value	0.59	<.0001	<.0001	<.0001	<.0001	<.0001	<.0001	0.95
Control – Minimal NfL	*MD*	0.04	-0.07	-0.02	0.07	-0.02	-0.04	-0.05	-0.03
	95% CI	(0.54, 0.59)	(-1.07, 0.28)	(-0.24, 0.81)	(0.95, 0.35)	(-0.32, 0.75)	(-0.54, 0.59)	(-0.65, 0.52)	(-0.41, 0.95)
	p-value	0.59	0.28	0.81	0.35	0.75	0.59	0.52	0.95

MD: Mean difference. CI: Confidence Interval. RPQ: Rivermead Post Concussion Questionnaire. NfL: Neurofilament light.

© 2024 O'Brien WT et al. *JAMA Network Open.*

eTable 9: Linear mixed models results for GFAP subgroup, NfL subgroup and loss of consciousness (LOC) as predictors of time to return to training.

Comparison	Time to Return to Training
GFAP extreme vs GFAP moderate	1.99 (1.69, 2.34) *p<0.001*
NfL extreme vs NfL minimal/no change	3.24 (2.63,3.97) *p<0.0001*
NfL moderate vs NfL minimal/no change	1.43 (1.18, 1.72) *p<0.0001*
LOC vs no LOC	1.65 (1.41,1.93) *p<0.0001*

Note: Return to training affected by end-of-season, COVID-19 lockdowns, or failure to return to training meant 15 cases were not included. Data is presented as incident rate ratio (95% confidence interval). GFAP: Glial fibrillary acidic protein. NfL: Neurofilament light. LOC: Loss of consciousness.

eTable 10: Post hoc results for comparisons of longitudinal outcomes in sport-related concussion (SRC) participants with and without loss of consciousness.

		24h	1w	2w	4w	6w	8w	12w	26w
**RPQ Symptom Score**									
No LOC - LOC	*MD*	-3.84	-2.68	-1.77	-0.68	-0.34	-0.35	0.24	1.35
	95% CI	(-7.07, -0.60)	(-5.48, 0.13)	(-4.55, 1.01)	(-3.38, 2.02)	(-2.89, 2.22)	(-3.14, 2.43)	(-2.50, 2.97)	(-1.55, 4.26)
	p-value	0.02	0.06	0.21	0.62	0.80	0.80	0.87	0.36
**Cogstate Detection Task (Latency) –** ***No significant interaction***									
**Cogstate Identification Task (Latency) –** ***No significant interaction***									
**Cogstate One Card Learning Task (Accuracy) –** ***No significant interaction***									
**Cogstate One Back Task (Latency) –** ***No significant interaction***									
**Serum GFAP**									
No LOC - LOC	*MD*	-1.01	-0.27	-0.21	-0.34	-0.16	-0.16	-0.009	-0.06
	95% CI	(-1.24, -0.77)	(-0.49, -0.06)	(-0.42, -0.004)	(-0.55, -0.13)	(-0.37, 0.05)	(-0.37, 0.05)	(-0.21, 0.19)	(-0.26, 0.15)
	p-value	<.0001	0.01	0.046	0.002	0.14	0.13	0.93	0.58
**Serum NfL**									
No LOC - LOC	*MD*	-0.20	-0.73	-0.91	-0.90	-0.81	-0.73	-0.54	-0.13
	95% CI	(-0.52, 0.11)	(-1.03, -0.42)	(-1.21, -0.61)	(-1.20, -0.59)	(-1.13, -0.50)	(-1.04, -0.42)	(-0.85, -0.22)	(-0.45, 0.19)
	p-value	0.20	<.0001	<.0001	<.0001	<.0001	<.0001	0.001	0.44

MD: Mean difference. CI: Confidence Interval. RPQ: Rivermead Post Concussion Questionnaire. GFAP: Glial fibrillary acidic protein. NfL: Neurofilament light. LOC: Loss of consciousness.

© 2024 O'Brien WT et al. *JAMA Network Open.*

eTable 11: Linear Mixed Model results for 24hr GFAP, 1w NfL and LOC as predictors of 24hr RPQ Symptom Severity and Time to Return to Training.

	IRR	95% Confidence Interval	p-value
24hr GFAP vs 24hr RPQ	1.01	(0.95, 1.08)	0.63
24hr GFAP* vs 24hr RPQ	1.01	(0.95, 1.08)	0.64
1w NfL vs 24hr RPQ	0.99	(0.92, 1.07)	0.77
1w NfL* vs 24hr RPQ	1.00	(0.92, 1.08)	0.99
LoC vs 24hr RPQ	1.18	(1.05, 1.32)	0.004
24hr GFAP vs Return to Training	1.40	(1.29, 1.52)	<0.001
24hr GFAP* vs Return to Training	1.41	(1.29, 1.53)	<0.001
1w NfL vs Return to Training	1.52	(1.41, 1.63)	<0.001
1w NfL* vs Return to Training	1.50	(1.40, 1.61)	<0.001
LoC vs Return to Training	1.54	(1.33, 1.79)	<0.001

Note: Return to training affected by end-of-season, COVID-19 lockdowns, or failure to return to training meant 15 cases were not included. *denotes values are adjusted for age and BMI. IRR: Incident Rate Ratio.

© 2024 O'Brien WT et al. *JAMA Network Open.*

eTable 12: Post hoc results for sensitivity analyses.

			24hr	1w	2w	4w	6w	8w	12w	26w
**GFAP SRC vs control**		*All Data*	**0.66 (0.50,0.82)**	0.03 (-0.12,0.19)	-0.003 (-0.15,0.15)	**0.17 (0.02,0.32)**	0.04 (-0.11,0.19)	-0.005 (-0.15,0.14)	-0.07 (-0.21,0.07)	-0.03 (-0.18,0.12)
		-12mo	**0.65 (0.49,0.81)**	0.002 (-0.16,0.16)	0.02 (-0.13,0.18)	0.14 (-0.01,0.30)	0.05 (-0.11,0.20)	0.002 (-0.15,0.15)	-0.07 (-0.22,0.07)	-0.03 (-0.18, 0.12)
		- x2	**0.62 (0.46,0.79)**	0.02 (-0.13,0.18)	-0.01 (-0.16, 0.14)	0.15 (-0.003, 0.30)	0.02 (-0.13,0.18)	-0.016 (-0.17,0.14)	-0.08 (-0.23,0.07)	-0.04 (-0.20,0.11)
		+ cov	**0.068 (0.51,0.84)**	0.05 (-0.10,0.21)	0.02 (-0.13,0.17)	**0.19 (0.03,0.34)**	0.06 (-0.09,0.21)	0.01 (-0.14,0.17)	-0.05 (-0.20,0.09)	-0.01 (-0.17,0.14)
**NfL SRC vs control**		*All Data*	0.07 (-0.14,0.27)	**0.31 (0.12,0.51)**	**0.38 (0.19,0.58)**	**0.31 (0.12,0.51)**	**0.27 (0.07,0.47)**	**0.36 (0.15,0.56)**	**0.25 (0.04,0.46)**	0.03 (-0.18,0.25)
		-12mo	0.05 (-0.16,0.25)	**0.30 (0.10,0.49)**	**0.36 (0.17,0.56)**	**0.29 (0.09,0.49)**	**0.25 (0.06,0.45)**	**0.34 (0.14,0.55)**	**0.23 (0.02,0.44)**	0.02 (-0.20,0.23)
		- x2	0.06 (-0.15,0.26)	**0.29 (0.10, 0.49)**	**0.35 (0.15,0.55)**	**0.27 (0.07,0.47)**	**0.24 (0.04,0.44)**	**0.33 (0.12, 0.54)**	**0.23 (0.02, 0.45)**	0.02 (-0.20,0.24)
		+ cov	0.09 (-0.12,0.30)	**0.34 (0.14,0.54)**	**0.41 (0.20,0.61)**	**0.33 (0.13,0.54)**	**0.30 (0.09,0.50)**	**0.38 (0.17,0.59)**	**0.27 (0.06,0.49)**	0.06 (-0.16,0.28)
**GFAP Extreme GFAP vs control**		*All Data*	**2.20 (1.89,2.50)**	**0.27 (-0.03,0.57)**	**0.39 (0.09,0.70)**	**0.48 (0.17,0.78)**	**0.29 (-0.02,0.59)**	0.10 (-0.21,0.40)	-0.04 (-0.35,0.26)	-0.14 (-0.45,0.17)
		-12mo	**1.94 (1.65,2.24)**	0.23 (-0.06,0.53)	**0.30 (0.003,0.59)**	0.38 (0.08,0.68)	0.18 (-0.12,0.48)	0.06 (-0.24,0.36)	-0.08 (-0.38,0.22)	-0.21 (-0.52,0.11)
		- x2	**1.79 (1.48,2.10)**	**0.28 (-0.02,0.59)**	0.17 (-0.13,0.46)	**0.51 (0.21,0.82)**	0.20 (-0.12,0.51)	0.12 (-0.20,0.44)	0.10 (-0.42,0.23)	0.18 (-0.51,0.15)
		+ cov	**2.20 (1.90,2.51)**	**0.28 (-0.03,0.58)**	**0.40 (0.09, 0.71)**	**0.48 (0.18,0.79)**	0.29 (-0.01,0.60)	0.10 (-0.20,0.41)	-0.04 (-0.34,0.27)	-0.13 (-0.45,0.18)

© 2024 O'Brien WT et al. *JAMA Network Open.*

eTable 12 (continued): Post-hoc results for secondary analyses.

		24hr	1w	2w	4w	6w	8w	12w	26w
**GFAP Moderate GFAP vs control**	*All Data*	**0.38 (0.20,0.55)**	-0.03 (-0.21,0.14)	-0.03 (-0.20,0.15)	0.10 (-0.07,0.28)	0.02 (-0.16,0.19)	-0.01 (-0.19,0.16)	-0.07 (-0.25,0.11)	-0.01 (-0.20,0.18)
	-12mo	**0.35 (0.17,0.53)**	-0.05 (-0.23,0.14)	-0.03 (-0.21,0.15)	0.09 (-0.09,0.28)	0.02 (-0.16,0.20)	-0.01 (-0.20,0.17)	-0.07 (-0.26,0.12)	-0.003 (-0.20,0.19)
	- x2	**0.38 (0.20, 0.57)**	-0.04 (-0.22, 0.14)	-0.06 (-0.23,0.12)	0.06 (-0.12, 0.25)	-0.02 (-0.21, 0.17)	-0.05 (-0.25, 0.14)	-0.09 (-0.29, 0.10)	0.03 (-0.23, 0.17)
	+ cov	**0.40 (0.23,0.58)**	-0.003 (-0.18,0.18)	0.002 (-0.18,0.18)	0.13 (-0.05,0.31)	0.04 (-0.14,0.23)	0.01 (-0.17,0.20)	-0.04 (-0.23,0.14)	0.02 (-0.18,0.21)
**NfL Extreme NfL vs control**	*All Data*	**0.68 (0.21,1.15)**	**2.40 (1.98,2.82)**	**2.72 (2.31,3.14)**	**2.46 (2.04,2.89)**	**2.48 (2.03,2.93)**	**2.23 (1.78,2.69)**	**1.73 (1.27,2.18)**	**0.84 (0.40,1.28)**
	-12mo	0.61 (-0.13,1.34)	**2.47 (1.75,3.18)**	**2.74 (2.03,3.44)**	**2.36 (1.64,3.09)**	**2.42 (1.68,3.15)**	**2.11 (1.37,2.85)**	**1.67 (0.93,2.40)**	**0.003 (-0.26,0.25)**
	- x2	**0.64 (0.12,1.17)**	**2.42 (1.95,2.88)**	**2.71 (2.26,3.16)**	**2.39 (1.92,2.86)**	**2.44 (1.94,2.93)**	**2.19 (1.69,2.69)**	**1.69 (1.19,2.19)**	**0.91 (0.43,1.40)**
	+ cov	**0.69 (0.20,1.18)**	**2.42 (1.98,2.87)**	**2.75 (2.32,3.18)**	**2.49 (2.05,2.92)**	**2.51 (2.06,2.96)**	**2.26 (1.81,2.71)**	**1.75 (1.31,2.19)**	**0.87 (0.46,1.27)**
**NfL Moderate NfL vs control**	*All Data*	0.08 (-0.25,0.41)	**0.82 (0.50,1.13)**	**0.96 (0.65,1.27)**	**0.84 (0.52,1.16)**	**0.87 (0.55,1.20)**	**0.86 (0.52,1.19)**	**0.59 (0.25,0.93)**	0.02 (-0.32,0.37)
	-12mo	0.014 (-0.39,0.41)	**0.74 (0.35,1.13)**	**0.88 (0.49,1.26)**	**0.76 (0.37,1.16)**	**0.80 (0.40,1.20)**	**0.79 (0.38,1.19)**	**0.52 (0.11,0.93)**	0.001 (-0.42,0.42)
	- x2	0.07 (-0.26,0.41)	**0.81 (0.49,1.13)**	**0.95 (0.63,1.26)**	**0.82 (0.50,1.15)**	**0.86 (0.53,1.19)**	**0.85 (0.51,1.19)**	**0.58 (0.24,0.93)**	0.02 (-0.33,0.37)
	+ cov	0.10 (-0.25,0.44)	**0.84 (0.50,1.17)**	**0.97 (0.65,1.30)**	**0.85 (0.52,1.18)**	**0.89 (0.56,1.22)**	**0.87 (0.54,1.20)**	**0.60 (0.27,0.93)**	0.04 (-0.28,0.36)

© 2024 O'Brien WT et al. *JAMA Network Open.*

eTable 12 (continued): Post-hoc results for secondary analyses.

		24hr	1w	2w	4w	6w	8w	12w	26w
**NfL No/minimal NfL vs control**	*All Data*	-0.04 (-0.59,0.54)	0.07 (-0.28,1.07)	0.02 (-0.81,0.24)	-0.07 (-0.35,0.95)	0.02 (-0.75,0.32)	0.04 (-0.59,0.54)	0.05 (-0.52,0.65)	0.03 (-0.95,0.41)
	-12mo	-0.11 (-0.35,0.13)	-0.004 (-0.23,0.23)	-0.06 (-0.28,0.17)	-0.13 (-0.37,0.10)	-0.04 (-0.28,0.20)	-0.02 (-0.26,0.22)	-0.01 (-0.26,0.23)	0.003 (-0.26,0.25)
	- x2	-0.05 (-0.24,0.15)	0.06 (-0.13,0.25)	0.001 (-0.18, 0.19)	0.09 (-0.28,0.11)	0.001 (-0.20, 0.20)	0.03 (-0.17, 0.23)	0.04 (-0.16, 0.25)	0.009 (-0.20, 0.22)
	+ cov	-0.02 (-0.22,0.18)	0.09 (-0.10,0.29)	0.04 (-0.16,0.23)	-0.05 (-0.24,0.15)	0.04 (-0.15,0.24)	0.06 (-0.14,0.25)	0.06 (-0.13,0.26)	0.04 (-0.15,0.24)
**GFAP LoC vs noLoC**	*All Data*	**1.01 (0.77,1.24)**	**0.27 (0.06,0.49)**	**0.21 (0.004,0.42)**	**0.34 (0.13,0.55)**	0.16 (-0.05,0.37)	0.16 (-0.05,0.37)	0.009 (-0.19,0.21)	0.06 (-0.15,0.26)
	-12mo	**0.92 (0.68,1.16)**	**0.26 (0.03,0.48)**	**0.19 (-0.03,0.41)**	**0.29 (0.08,0.51)**	0.14 (-0.08,0.36)	0.13 (-0.08,0.35)	0.01 (-0.20,0.22)	0.04 (-0.17,0.26)
	- x2	**0.93 (0.70,1.17)**	**0.25 (0.03,0.47)**	0.17 (-0.04, 0.39)	**0.29 (0.07,0.50)**	0.14 (-0.07,0.36)	0.15 (-0.06,0.37)	0.01 (-0.20,0.22)	0.03 (-0.19,0.24)
	+ cov	**0.99 (0.75,1.23)**	**0.26 (0.04,0.49)**	**0.20 (-0.01,0.42)**	**0.33 (0.11,0.55)**	0.15 (-0.07,0.47)	0.15 (-0.06,0.37)	0.002 (-0.21,0.21)	0.05 (-0.16,0.26)
**NfL LoC vs noLoC**	*All Data*	0.20 (-0.11,0.52)	**0.73 (0.42,1.03)**	**0.91 (0.61,1.21)**	**0.90 (0.59,1.20)**	**0.81 (0.50,1.13)**	**0.73 (0.42,1.04)**	**0.54 (0.22,0.85)**	0.13 (-0.19,0.45)
	-12mo	0.23 (-0.12,0.59)	**0.69 (0.34,1.03)**	**0.86 (0.52,1.19)**	**0.85 (0.52,1.18)**	**0.76 (0.43,1.09)**	**0.69 (0.37,1.00)**	**0.50 (0.20,0.80)**	0.18 (-0.07,0.43)
	- x2	0.21 (-0.13,0.55)	**0.69 (0.36,1.02)**	**0.85 (0.53,1.18)**	**0.84 (0.52,1.15)**	**0.76 (0.44,1.08)**	**0.69 (0.38,1.00)**	**0.49 (0.20,0.79)**	0.16 (-0.08,0.40)
	+ cov	0.22 (-0.11,0.54)	**0.71 (0.40,1.02)**	**0.92 (0.61,1.23)**	**0.92 (0.61,1.23)**	**0.79 (0.48,1.11)**	**0.75 (0.42,1.07)**	**0.54 (0.22,0.87)**	0.13 (-0.20,0.46)

Sensitivity analyses were performed excluding the eight participants that had sustained a SRC in the previous 12 months *(-12mo*), the nine participants that participated twice in the study *(-x2*), and the inclusion of number of concussions, sex and psychiatric disease as additional covariates (+cov). Post-hoc results for the primary group (*all data)* comparisons are provided as a point of reference. Data is presented as mean difference (95% confidence intervals) and p-values<0.05 are bolded.

© 2024 O'Brien WT et al. *JAMA Network Open.*
