# Supplementary material for: Biomarkers of Neurobiologic Recovery in Adults With Sport-Related Concussion
Source: JAMA Netw Open. 2024 Jun 7;7(6):e2415983. doi: 10.1001/jamanetworkopen.2024.15983 (PMC11161851; doi:10.1001/jamanetworkopen.2024.15983)
Supplement: Supplement 2. — Data Sharing Statement [file jamanetwopen-e2415983-s002.pdf]

Data Sharing Statement

O'Brien. Serum Glial Fibrillary Acidic Protein and Neurofilament Light Trajectories in Adults With Sport-Related Concussion. *JAMA Netw Open*. Published June 03, 2024.
doi:10.1001/jamanetworkopen.2024.15983

Data
Data available: Yes
Data types: Deidentified participant data
How to access data: stuart.mcdonald@monash.edu
When available: With publication

Supporting Documents
Document types: None

Additional Information
Who can access the data: Data will be made available by the corresponding author upon reasonable request.
Types of analyses: Data may be made available on request for any scientific purpose.
Mechanisms of data availability: Data may be made available after approval of a proposal and with signed data access agreement.
